# The Eyes Have It: Regulatory and Structural Changes Both Underlie Cichlid Visual Pigment Diversity

**DOI:** 10.1371/journal.pbio.1000266

**Published:** 2009-12-22

**Authors:** Christopher M. Hofmann, Kelly E. O'Quin, N. Justin Marshall, Thomas W. Cronin, Ole Seehausen, Karen L. Carleton

**Affiliations:** 1 Department of Biology, University of Maryland, College Park, Maryland, United States of America; 2 Sensory Neurobiology Group, School of Biomedical Sciences University of Queensland, St. Lucia, Queensland, Australia; 3 Department of Biological Sciences, University of Maryland Baltimore County, Baltimore, Maryland, United States of America; 4 Aquatic Ecology and Evolution, Institute of Ecology and Evolution, University of Bern, Bern, Switzerland; 5 Eawag, Swiss Federal Institute for Aquatic Science and Technology, Centre of Ecology, Evolution and Biogeochemistry, Kastanienbaum, Switzerland; Duke University, United States of America

## Abstract

A major goal of evolutionary biology is to unravel the molecular genetic mechanisms that underlie functional diversification and adaptation. We investigated how changes in gene regulation and coding sequence contribute to sensory diversification in two replicate radiations of cichlid fishes. In the clear waters of Lake Malawi, differential opsin expression generates diverse visual systems, with sensitivities extending from the ultraviolet to the red regions of the spectrum. These sensitivities fall into three distinct clusters and are correlated with foraging habits. In the turbid waters of Lake Victoria, visual sensitivity is constrained to longer wavelengths, and opsin expression is correlated with ambient light. In addition to regulatory changes, we found that the opsins coding for the shortest- and longest-wavelength visual pigments have elevated numbers of potentially functional substitutions. Thus, we present a model of sensory evolution in which both molecular genetic mechanisms work in concert. Changes in gene expression generate large shifts in visual pigment sensitivity across the collective opsin spectral range, but changes in coding sequence appear to fine-tune visual pigment sensitivity at the short- and long-wavelength ends of this range, where differential opsin expression can no longer extend visual pigment sensitivity.

## Abstract 2

Differential gene expression and coding sequence evolution play complementary roles in the adaptive diversification of cichlid sensory systems.

## Author Summary

The molecular mechanisms that generate biodiversity remain largely elusive. We examined how two of these mechanisms, changes in gene expression and changes in gene coding sequence, have generated an incredibly diverse set of visual systems in rapidly speciating African cichlids. We found large differences in cone opsin gene expression among cichlids inhabiting the clear waters of Lake Malawi. These changes are likely to have strong influences on retinal sensitivity and appear to be driven primarily by different foraging needs. Cichlids inhabiting the turbid waters of Lake Victoria, however, only expressed a subset of their opsin genes and variation in gene expression appears to by driven primarily by the spectrum of environmental light. When we compared the sequences of these opsin genes, we found greater variation in the genes at the ultraviolet and red edges of the sensitivity range. Taken together these findings suggest that changes in gene expression and coding sequence can be complementary and work in concert to generate changes in sensory systems. Because of their correlation with ecological factors, these changes are also likely to be adaptive and to have played a role in generating the tremendous diversity of cichlids in these two lakes.

## Introduction

A very large body of literature has been dedicated to the geography, ecology, and genetics of adaptive diversification and speciation [1]–[5]. Yet, the proximate mechanisms responsible for diversification have been characterized for only a few traits in a few systems [3]. The molecular genetic mechanisms underlying functional diversification can be divided into two major categories. First, changes in gene expression (either through *cis*- or *trans*-acting regulatory factors) can alter the type, location, timing, or amount of protein produced. Alternatively, changes in gene coding sequence can alter protein function. The relative contributions of these mechanisms have been debated since King and Wilson proposed that functional species differences are largely the result of differential gene expression [6]. Recent studies have confirmed the key role that altered gene expression plays in modifying body form or pattern (e.g., [7]–[10]). However, structural changes in proteins also contribute to phenotypic adaptation (e.g., [11]–[14]). Recently, sweeping claims regarding the importance of each mechanism have been made by proponents on both sides of the debate [14],[15], whereas others have argued that this dichotomy is arbitrary [16],[17]. In spite of this debate, few studies have examined the relative role that both mechanisms can play in shaping a single phenotype.

The visual system is ideal for investigating the molecular mechanisms of adaptation, because there is a direct link between genotype and phenotype [18],[19]. Within the retina, spectral sensitivity is determined by visual pigments, which are composed of an opsin protein bound to a light-sensitive chromophore [20]. This opsin–chromophore interaction determines the peak spectral sensitivity of each visual pigment. Numerous studies have demonstrated that visual pigment sensitivities are tuned to the local light environment by amino acid substitutions in opsin proteins [12],[18],[19],[21]–[26]. Consequently, sensory adaptation via changes in opsin gene coding sequence has become a classic example of molecular adaptation.

However, fish have numerous opsin genes that have arisen through tandem gene duplications. These duplicate opsin genes have diverged to produce visual pigments that absorb maximally across the full spectral range, from the ultraviolet to the red (reviewed in [27]). Recent work in cichlids and other taxa has demonstrated that differential expression of these opsin genes may generate large changes in visual sensitivity [28]–[31]. Typically, these studies have examined populations of one species, or of closely related species, but have not evaluated the relative importance, and adaptive significance, of spectral tuning via differential gene expression across many divergent species.

The haplochromine cichlids of the East African rift lakes are well suited for addressing this question. They are a classic example of adaptive radiation and rapid speciation [2],[32]–[36]. Hundreds of new species have evolved in Lake Malawi within the past 1–2 million years and within a mere 15,000–120,000 years in Lake Victoria [37],[38]. These two haplochromine radiations provide a large number of closely related, yet ecologically and morphologically divergent, species. Furthermore, these two lakes differ dramatically in their light environment [39]. Lake Malawi is one of the deepest and clearest freshwater lakes in the world, with clarity similar to that of marine environments [40]. In contrast, Lake Victoria is relatively turbid, with long wavelength–shifted transmission and considerable variation in both clarity and transmission among geographic localities [41]. Studies have demonstrated repeatedly that selection is acting on the visual systems of cichlids in both lakes [22]–[26],[42],[43].

In this paper, we use these two replicate cichlid radiations to (1) examine how changes in opsin gene expression contribute to the remarkable diversification of cichlid visual systems, (2) test whether changes in opsin gene expression are adaptive, and (3) compare the relative roles that differential opsin gene expression and changes in protein coding sequence play in the diversification of cichlid visual systems.

## Results

### Opsin Expression Profiles

We quantified opsin gene expression in 54 wild-caught taxa from Lake Malawi and 11 lab-reared taxa from Lake Victoria (Tables S1 and S2). Cichlids have one rod opsin gene (*Rh1*) and six functionally and genetically distinct classes of cone opsin: *SWS1* (ultraviolet, or UV), *SWS2B* (violet), *SWS2A* (blue), *Rh2B* (blue-green), *Rh2A* (green), and *LWS* (red) [29],[30],[44]. (As in previous cichlid studies, we group expression of the functionally and genetically similar *Rh2Aα* and *Rh2Aβ* together [25],[29],[30].) Cichlid retinas are highly organized, and the shorter-wavelength *SWS* opsins are expressed in morphologically distinct single cones, whereas the longer-wavelength *Rh2* and *LWS* genes are expressed in double cones [25],[30],[44],[45].

**Figure 1:**
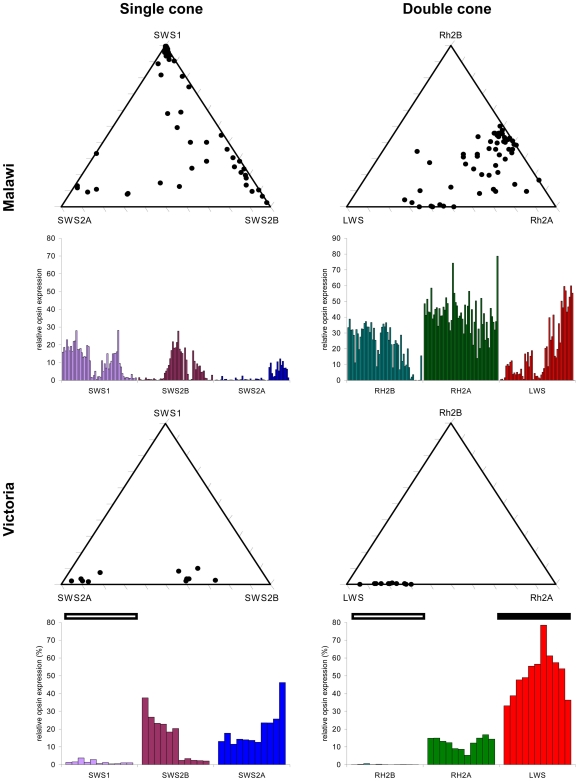
Opsin gene expression from all species surveyed. Triangle plots illustrate the relationships between opsins within the single and double cones of Lake Malawi and Victoria cichlids. Malawian single and double cones vary along two axes, whereas Victorian single and double cones only vary along one. Bar graphs below each plot show expression of the corresponding opsins and emphasize the qualitative differences in expression profiles between lakes. No Lake Victoria taxa express more than trace amounts of *SWS1* or *Rh2B* (open bar), and all express high levels of *LWS* (filled bar). Opsin expression was measured using real-time PCR. Each point or column represents a different taxon (see Table S1). Triangle plots were generated using a freely available Excel worksheet [74].

**Figure 2:**
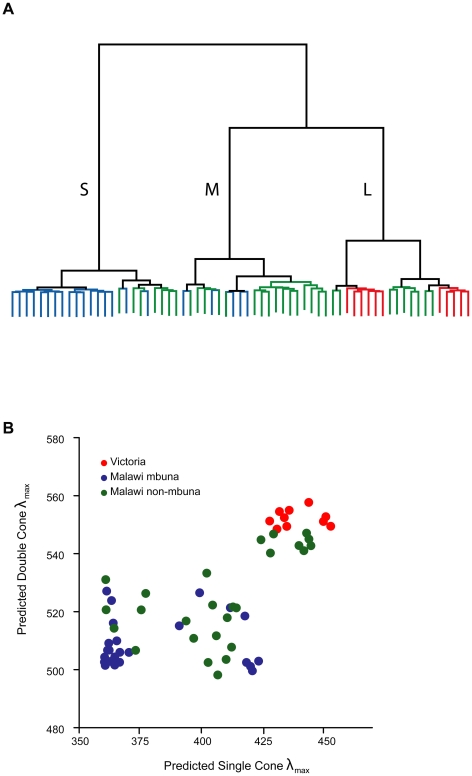
Gene expression profiles and single- and double-cone sensitivities form three clusters. (A) Hierarchical clustering of species' opsin expression profiles revealed three clusters. S, short wavelength; M, medium wavelength; L, long wavelength. (B) Estimates of Lake Malawi single- and double-cone sensitivities suggest that these three clusters correspond to visual palettes sensitive to short-, medium-, and long-wavelength portions of the cichlid visible light spectrum. Species from the mbuna clade are shown in blue, non-mbuna are shown in green. Lake Victoria cichlids (red) all fall within the longest-wavelength Malawian cluster. Single- and double-cone λ_max_ values were estimated by weighting the peak absorbance of each opsin by its relative expression level.

Cichlids from Lake Malawi had diverse expression profiles that collectively expressed all six cone opsin genes (Figure 1). These expression profiles formed three distinct clusters (Figure 2A) with support based on multiple cluster validation statistics (Table S8). Members of the mbuna clade predominantly expressed the shorter-wavelength classes of opsin genes: all species sampled expressed *SWS1* or *SWS2B* opsins in their single cones, and fewer than half of these species (12/26) expressed the longer-wavelength *LWS* opsin in their double cones. Non-mbuna collectively expressed all three *SWS* opsins in their single cones, although the overwhelming majority of the species sampled (23/26) expressed *LWS* in their double cones (Table S1). In both lineages, we found examples of closely related species that expressed different subsets of opsin genes, suggesting that sister taxa could differ significantly in visual sensitivity (Figure S1). Such differences occurred in 12 of the 14 genera in which we sampled multiple species, and included genera as diverse as *Tropheops*, *Melanochromis*, *Protomelas*, *Dimidiochromis*, and *Rhamphochromis*.

Cichlids inhabiting Lake Victoria collectively expressed four different opsin classes (Figure 1), and their expression profiles fell within a single cluster (Figure 2A). None of the taxa that we examined expressed more than trace amounts of *SWS1* or *Rh2B*. All of the Victorian species expressed *SWS2A* in their single cones and *Rh2A* and *LWS* in their double cones. Several taxa also expressed *SWS2B* in their single cones, and *SWS2B* expression was variable, even among conspecifics from different geographic localities (rocky islands). We therefore treated each localized population as a distinct group in subsequent analyses (Table S1).

To examine how changes in gene expression might shape overall retinal sensitivity, we used data from reconstituted cichlid visual pigments [29] to estimate average single- and double-cone sensitivities for each species [30]. The estimated single- and double-cone sensitivities of Malawian taxa fell into three distinct groups sensitive to short-, middle-, and long-wavelength regions of the spectrum (Figure 2B). These groups correspond directly to the gene expression clusters (Figure 2A) and were also supported by multiple cluster validation statistics (Table S8). Although there was some variation in single- and double-cone sensitivities within Lake Victoria, all Victorian taxa fell into the long-wavelength group.

### Ecological Factors Driving Divergent Opsin Expression

**Figure 3:**
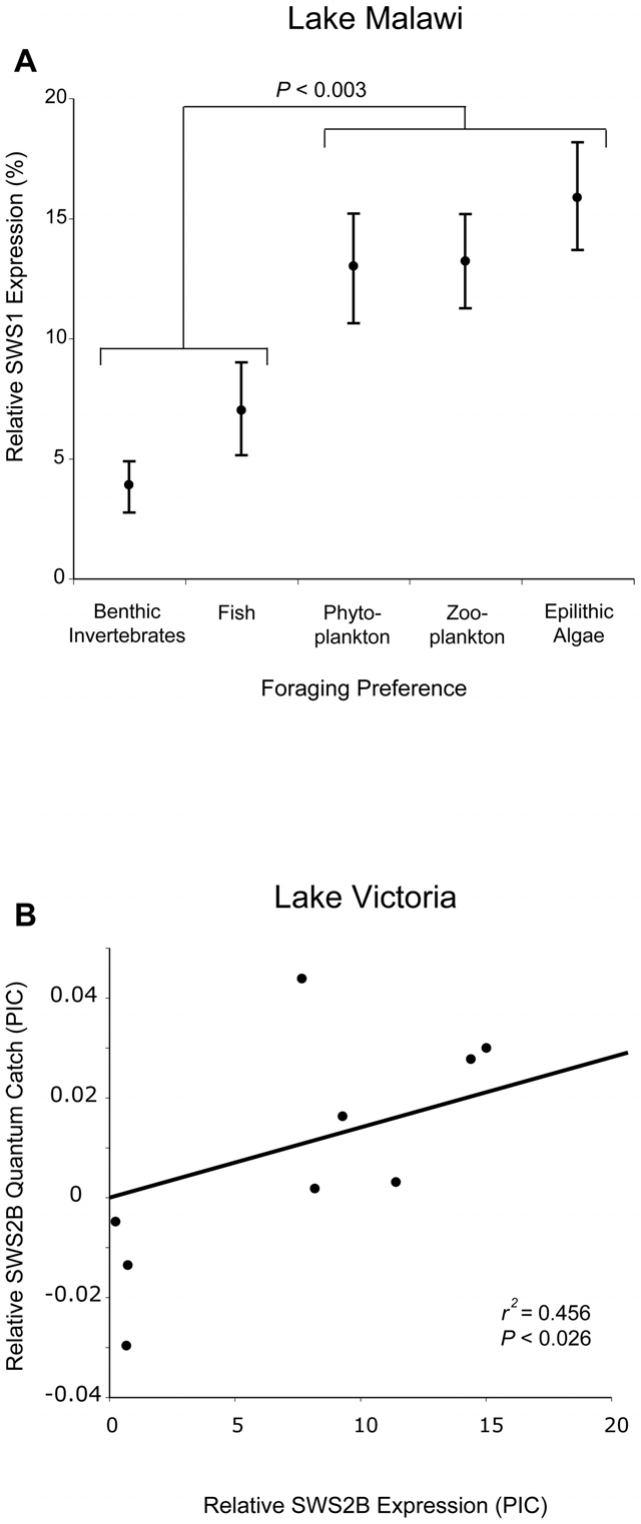
Selective pressures drive opsin expression within each lake. (A) Relative *SWS1* (ultraviolet) opsin expression is higher among Lake Malawi cichlids foraging on phytoplankton, zooplankton, and epilithic algae (phylogenetic ANOVA). (B) Relative *SWS2B* opsin expression is positively correlated with the predicted quantum catch that an SWS2B-based visual pigment would have at clear and murky locations in Lake Victoria (phylogenetically independent contrasts [PIC]). See Figure S2 for the phylogenies used in these comparative analyses.

To test whether changes in gene expression were adaptive, we compared mean opsin expression and estimated photoreceptor sensitivity among cichlids with different foraging and habitat preferences. Using phylogenetically controlled comparative methods, we found that the *SWS1* opsin gene was differentially expressed among Lake Malawi cichlids with different foraging preferences (phylogenetic ANOVA, *F*_4,45_ = 7.647, *p* = 0.007, Table S3). *SWS1* expression was highest among species foraging on zooplankton, phytoplankton, and algae, and lowest among species foraging on fish or benthic invertebrates (*F*_1,52_ = 23.91, *p* = 0.003, Figure 3A). Up-regulation of *SWS1* also resulted in estimated single-cone sensitivities that differed among these species (phylogenetic ANOVA, *F*_4,45_ = 9.065, *p* = 0.002). Cichlids foraging on plankton and algae typically exhibited single-cone sensitivities peaking between 360 and 400 nm, such that they would be more sensitive to UV light than either piscivores or benthivores. *SWS1* was the only opsin significantly associated with foraging preferences. We did not observe significant differences in opsin gene expression or single- and double-cone sensitivities among cichlids from different habitats (rock, sand, intermediate, pelagic, and weeds; Table S3).

Although we sampled Victorian taxa with a similar diversity of foraging preferences (e.g., planktivores, algivores, benthic foragers, and piscivores; Table S1), there was a complete absence of *SWS1* opsin expression among these cichlids, and all taxa fell into a single expression cluster. These findings suggest that foraging preferences are not likely to be a major driver of opsin expression in the Victorian species that we sampled. However, photic environment is known to influence visual sensitivities among populations and species of cichlids from this lake [24]–[26]. Therefore, we examined whether variation in the light environment between sampling sites could explain the pattern of gene expression that we observed.

**Figure 4:**
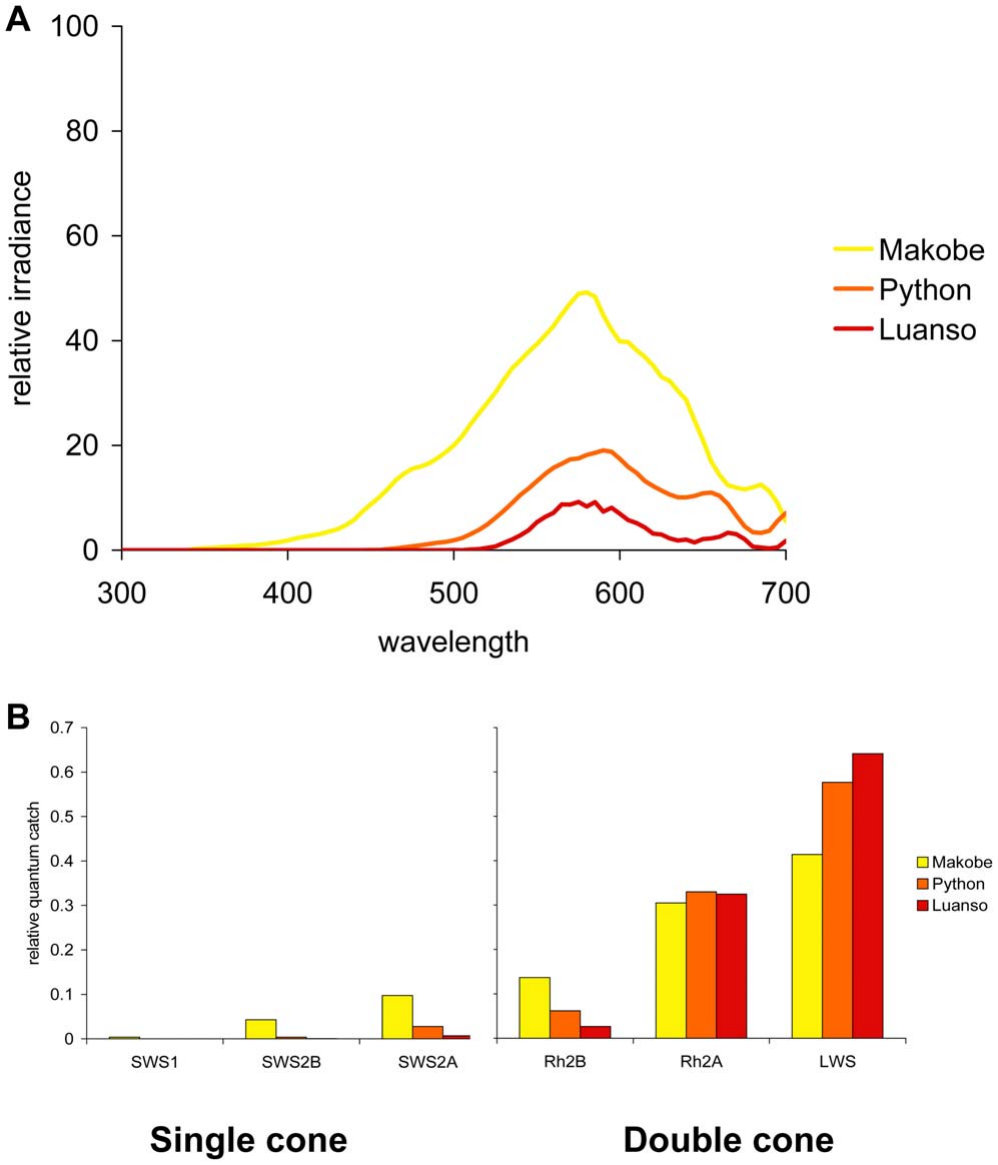
Visual pigment performance in Lake Victoria. (A) Relative irradiance at 2 m depth at three locations in Lake Victoria (Makobe Island, Python Island, and Luanso Island). (B) In Lake Victoria, estimated quantum catches are predicted to vary over several orders of magnitude, both across visual pigments and geographic locations.

We measured light transmission at three representative localities in Lake Victoria. We found that there was considerable variation between localities, with transmission decreasing and shifting to longer (redder) wavelengths from the open water site of Makobe to the sites of Python and Luanso, which were increasingly farther up the inlet of the Mwanza Gulf (Figure 4A). We then calculated how much of the available light a visual pigment composed of each opsin protein would capture at these different locations. In these spectrally narrow waters, quantum catches varied by almost four orders of magnitude (Figure 4B). SWS2A- and LWS-based visual pigments were predicted to have the greatest quantum catch in the single and double cones, respectively, whereas SWS1-based visual pigments would have virtually no quantum catch (Figure 4B). SWS2B-based visual pigments would capture some of the available light in the relatively clear waters of Makobe, but very little at the other two, more turbid locations.

Finally, we used water clarity and population-specific depth preferences to predict the quantum catch that an SWS2B-based visual pigment would have at the site where each taxon was originally sampled (Tables S1 and S4). We found that *SWS2B* opsin gene expression was positively correlated with predicted quantum catch (Figure 3B, Felsenstein's independent contrasts, *r* ^2^ = 0.456, *F*_1,4_ = 7.543, *p* = 0.023), suggesting that *SWS2B* expression is increased in environments where it is predicted to capture more of the available light.

**Figure 5:**
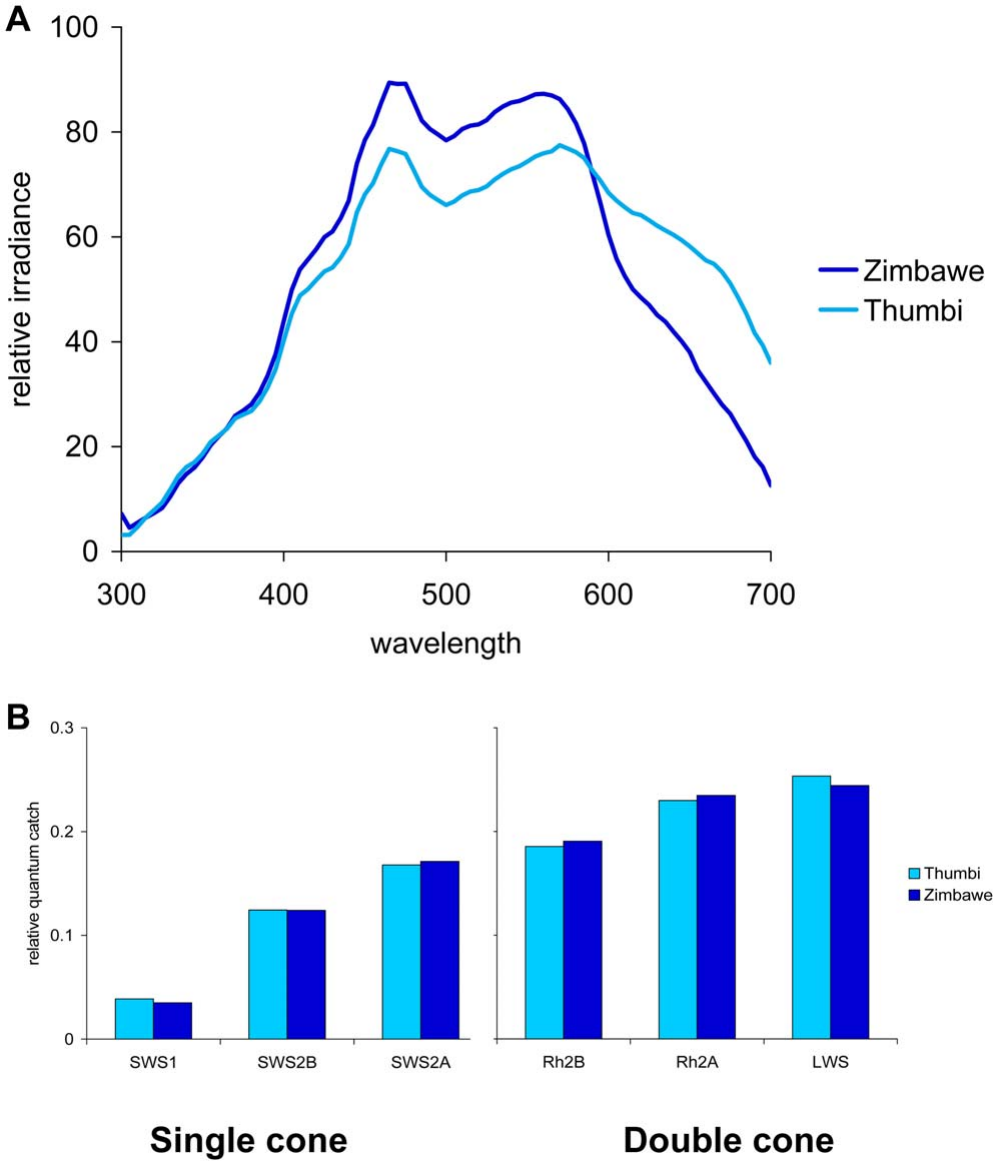
Visual pigment performance in Lake Malawi. (A) Relative irradiance at 2 m depth at two locations in Lake Malawi (Thumbi West Island and Zimbawe Rock). (B) In Lake Malawi, all visual pigments would have relatively similar, high quantum catches at both locations.

In the spectrally broad and relatively homogenous environment of Lake Malawi (Figure 5A), the estimated quantum catches do not vary appreciably between the two locations that we sampled (Zimbawe Rock, a deep, open-water site, and Thumbi West Island, a sheltered bay). Further, quantum catches vary by less than a single order of magnitude across opsin classes (Figure 5B). This finding suggests that environmental light is not likely to be a major driver of opsin gene expression in the species that were sampled from Lake Malawi.

### Changes in Opsin Coding Sequence

**Figure 6:**
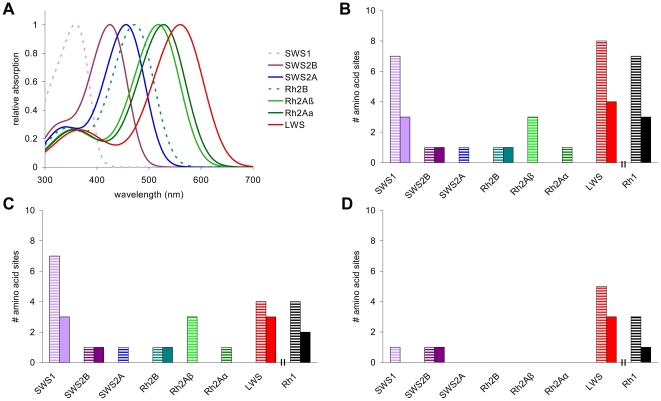
The shortest- and longest-wavelength opsins have the greatest sequence diversity. (A) Normalized absorbance values of all seven cichlid cone visual pigments. Curves were generated using the λ_max_ values from *O. niloticus* [29] and the equations of Govardovskii, et al. [68]. Dotted lines represent opsins that were not expressed in our Lake Victoria cichlid populations. (B) Number of amino acid substitutions in the transmembrane regions (barred) and retinal binding pocket (solid) regions of each opsin class from all species surveyed. (C) Number of substitutions in Lake Malawi. (D) Number of substitutions in Lake Victoria. Only substitutions between residues with different chemical properties are shown.

Several previous studies have documented the action of selection on different cichlid opsin genes [23]–[26],[42],[43]. To complement those studies, we compared coding sequence diversity across the cone and rod opsins of ten species from Lake Victoria and 16 species from Lake Malawi (Table S5). We focused on substitutions between amino acids with different chemical properties in the transmembrane and retinal binding pocket regions of the protein because changes in these regions are most likely to alter visual pigment sensitivity. We found that the number and nature of amino acid substitutions varied considerably across opsin classes (Figure 6B). Among species sampled from Lake Malawi (Figure 6C), the greatest diversity of functionally critical sites was found in the *SWS1* opsin, which had seven variable transmembrane sites, of which three were in the retinal binding pocket. Both the *LWS* and *Rh1* opsins exhibited four variable transmembrane sites, of which three and two, respectively, were in the retinal binding pocket. Among cichlids from Lake Victoria (Figure 6D), the number of functionally important sites was highest for the *LWS* opsin, which had five variable transmembrane sites, of which three were in the retinal binding pocket. Several of these substitutions were at sites that have been demonstrated previously to shift the spectral sensitivities of visual pigments (Table S6, Text S1). Longer wavelength shifts occur in species which inhabit deeper waters where the light is relatively more red-shifted [26]. The observed number of functional substitutions was independent of the number of synonymous changes and of overall nucleotide diversity (Figure S3).

## Discussion

We present a comprehensive analysis of opsin gene expression in over 60 different species of cichlids from Lakes Malawi and Victoria. We found that changes in opsin expression can generate diverse sets of visual systems. We also demonstrated that these changes in gene expression are adaptive and are shaped by foraging preferences and the local light environment. In addition, we examined coding sequence variation across the full complement of opsin genes. We found that diversity in functionally important regions is not distributed equally. Instead, diversity is highest in the opsin genes that code for the shortest- and longest-wavelength visual pigments. Although numerous studies have demonstrated the importance of changes in opsin coding sequence to visual adaptation in cichlids, only one study addressed adaptive changes in opsin gene expression, and this was only for a limited number of closely related species [25]. Our results suggest a model of sensory adaptation where evolutionary changes in both expression and coding sequence work in concert to shape visual pigment sensitivity.

### Visual System Diversity

We found that cichlids inhabiting the spectrally broad light environment of Lake Malawi had remarkable visual diversity and collectively expressed all six cone opsin genes. Although opsin expression was labile and could differ among closely related species, some structure emerged when the two major lineages within Lake Malawi were compared. Members of the mbuna or rock-dwelling clade predominantly expressed the shorter-wavelength classes of opsin genes in both single and double cones. Non-mbuna (sand-dwelling or pelagic species) collectively expressed all six opsins, but the middle- and longer-wavelength classes were predominant. Cichlids inhabiting the turbid waters of Lake Victoria express only four different classes of cone opsin. The shortest-wavelength single- and double-cone opsin genes were never expressed, and the longest-wavelength genes were expressed ubiquitously.

When we estimated single- and double-cone sensitivities based on patterns of opsin expression, we found that the species fell into three distinct short-, middle-, and long-wavelength clusters. These clusters correspond well with the three “visual palettes” documented previously in these and other cichlid species using microspectrophotometry (MSP) [30],[44],[46],[47]. Cichlids from Lake Malawi utilized every visual palette, whereas all Victorian cichlids grouped with the Malawian long-wavelength one. Thus, our results suggest that regulatory changes in opsin gene expression have generated diverse sets of single- and double-cone sensitivities. This extent of visual diversity among so many closely related species is extraordinary.

### Divergence in Opsin Expression Is Adaptive

We found evidence that changes in gene expression contributed to sensory adaptation, both to enhance foraging and to adapt to differences in the photic environment. The *SWS1* opsin gene, which encodes a UV-sensitive visual pigment, was differentially expressed between cichlids from different trophic groups in the clear waters of Lake Malawi. Species feeding on plankton or algae typically exhibited single-cone sensitivities peaking at shorter wavelengths than piscivores or benthic foragers. Studies of several teleost species, including two of the cichlids examined in this study, have demonstrated that UV sensitivity can increase the efficiency of foraging on zooplankton and other small organisms [48]–[50]. Additionally, many cichlids are opportunistic feeders, and several species have been observed to switch from foraging on algae to foraging on zooplankton or phytoplankton [51]. We found that expression of the *SWS1* opsin is highest precisely among cichlids foraging on these food sources (Figure 3A). Given that our comparative results are also supported by experimental and observational data, we believe that the observed differences in *SWS1* opsin expression are adaptive and that foraging may be a key driver of visual pigment diversity in Lake Malawi [52],[53].

Ambient light appears to have a strong influence on opsin expression in the spectrally narrow, longer-wavelength waters of Lake Victoria. We found that all of the Victorian species that we sampled exhibited similar expression profiles, with some variation in the expression of *SWS2B*. The predominant opsin genes expressed among these taxa—*SWS2A* (blue) in single cones, and *Rh2A* (green) and *LWS* (red) in double cones—were predicted to produce visual pigments with the greatest quantum catches in all three of our representative light environments. However, our predictions also suggested that an SWS2B-based visual pigment (violet) would capture some of the available light in clear locations, but much less in turbid ones. *SWS2B* opsin gene expression varied across taxa, and this variation was positively correlated with predicted quantum catch. Taken together, our findings suggest that ambient light is driving opsin gene expression in Lake Victoria.

One potential limitation of our study was that the Malawian samples were wild-caught, whereas the Victorian samples were lab-reared in a common garden environment. Although lab rearing and light manipulations have been demonstrated to alter levels of opsin expression, photoreceptor abundance, and photoreceptor length [31],[54]–[56], several lines of evidence suggest there is a large genetic component to opsin expression in cichlids. First, all three opsin expression clusters are observed in species raised in a common lab environment. In fact, the three opsin palettes of Lake Malawi were originally identified in lab-reared fish [28],[44], and all seven opsin genes are turned on in ontogenetic sequence in tilapia raised under laboratory conditions [29]–[30]. Second, genetic crosses between cichlid species with different visual palettes found a significant genetic component to opsin expression (K. L. Carleton, C. M. Hofmann, Klisz C, Z. Patel, L. M. Chircus, et al., unpublished data). Finally, direct comparisons of gene expression from wild-caught and lab-reared F_1_ fish from the same populations in Lake Malawi suggest that whereas levels of gene expression may change for some opsins in some species, expression of the shortest-wavelength *SWS1* and *SWS2B* opsins is maintained in the lab (C. M. Hofmann, K. E. O'Quin, A. R. Smith, K. L. Carleton, unpublished data). In sum, we feel that the lab rearing of Victorian samples is unlikely to influence our overall finding that differences in gene expression are adaptive.

### Potential for Speciation

The rapid changes in opsin gene expression that we observed among these closely related cichlid species are unprecedented in vertebrates. Differential gene expression among these species produces large shifts in spectral sensitivities (up to 100 nm) that could modify a species' view of conspecifics or the natural scene, and so modify species behavior. In Lake Victoria, changes in the coding sequence of the *LWS* opsin result in smaller shifts (5–15 nm) in visual pigment sensitivity that are linked to differences in depth, water clarity, and male color [24]–[26]. As a result, the *LWS* opsin gene is under strong selection and was shown recently to play a role in speciation in Victorian cichlids [26]. Since these fine-scale changes are linked to speciation, it is likely that the large differences in visual pigment sensitivity generated through differential opsin expression could also play such a role in cichlids from both lakes.

### Increased Diversity in the Longest- and Shortest-Wavelength Opsins

Opsin genes provide a clear example of how gene duplication and divergence in coding sequence can generate functional diversity in an adaptive phenotype [57]. We found strong evidence for functional coding differences among species, though these were not distributed equally across the opsins. The greatest number of functional coding differences were in the cone opsin genes that produce visual pigments at the ends of the cichlid visual range—the *SWS1* (UV) and *LWS* (red) opsins—as well as in the *Rh1* (rod) opsin. Since the rod opsin is the only opsin expressed in cichlid rods, rods cannot use the mechanism of differential gene expression to tune visual pigment sensitivity. Likewise, differential gene expression cannot extend spectral sensitivity beyond the boundaries set by the opsin genes that encode the shortest- and longest-wavelength visual pigments (because there are no shorter- or longer-wavelength genes to turn on). Therefore, all three of these genes must utilize coding sequence changes to alter visual pigment sensitivity. This pattern of sequence diversity is consistent with previous evidence that selection is acting on these three opsin genes [23],[24],[42],[43].

### A Model of Sensory Diversification

In this study, we examined the different contributions that changes in gene expression and coding sequence make to the diversification of cichlid visual systems. Our results suggest a model in which both proximate mechanisms contribute to visual pigment diversity. This model contains three main features: (1) Differential gene expression can generate large shifts in visual pigment sensitivity (30–100 nm) across the combined opsin spectral range. (2) Coding sequence substitutions fine-tune visual pigment sensitivity (5–15 nm) around each opsin's ancestral sensitivity. (3) Changes in coding sequence are more prevalent in the opsins operating at the short- and long-wavelength ends of the visual range, where differential gene expression can no longer extend visual pigment sensitivity. Therefore, although tuning in the middle portion of the visible-light spectrum is achieved by shifts in opsin gene expression, tuning at the ends of the visible light spectrum is achieved via opsin sequence evolution.

This model suggests that changes in gene expression and changes in protein coding sequence work in concert to generate phenotypic diversity. The extent to which our model can be applied to the visual systems of other teleosts, other sensory systems, or other genetic pathways remains to be seen. However, we predict that phenotypes influenced by multiple paralogous genes are likely to show similar patterns of expression and coding sequence evolution. We are currently examining the visual systems of Lake Tanganyika cichlids and damselfish. These two radiations are older than those in this study by one and two orders of magnitude, respectively, and will provide further tests for how coding sequence and gene expression interact in shaping visual phenotypes. Finally, we are performing genetic crosses to identify the specific loci that are responsible for the changes in gene expression that we observe. Understanding the timescales over which structural and regulatory changes act, and understanding the loci underlying regulatory changes, will provide further insights into when and how they work in concert to generate adaptive phenotypic change.

## Materials and Methods

### Ethics Statement

Fish were euthanized according to University of Maryland Institutional Animal Care and Use Committee (IACUC)-approved protocol (R-09-73).

### Opsin Gene Expression

We quantified relative opsin gene expression from 26 mbuna and 26 non-mbuna (*n* = 1–6 individuals per taxon) that were captured in the southern portion of Lake Malawi in 2005 from the south side of Thumbi West Island or off Otter Point. We also measured gene expression from 11 Victorian taxa (*n* = 1–5 individuals per taxon) from four different genera with diverse foraging modes and habitats (Table S1). Victorian fish were lab bred from wild-caught stocks and reared in a common garden laboratory environment at the Centre of Ecology, Evolution & Biogeochemistry of the ETH Institute for Aquatic Research in Kastanienbaum, Switzerland. Tanks were illuminated using daylight fluorescent light with a 12∶12 light∶dark cycle. Water temperature was kept constant at 24–26°C. All fish were raised on a mix of commercial flake food, given daily, and a blend of shrimp, peas, and *Spirulina* powder fed two times a week. Experimental tanks were part of a large recirculation system. All fish were sampled upon sexual maturity.

Fish were euthanized and retinas were dissected from the eyecup and immediately stored in RNAlater (Ambion) until the time of analysis. Retinas were collected from adult fish, greater than 6 mo of age, when any ontogenetic changes would be complete [30]. These were collected during the late morning through the afternoon. Although cichlid opsin gene expression does show diurnal variation, expression of cone opsin genes varies slowly and in synchrony [58]. Therefore, sampling time is not likely to impact the relative gene expression ratios we determined here.

Real-time PCR methods follow those previously optimized for cichlid opsins [28],[29]. In brief, RNA was extracted using commercially available kits (RNeasy, Qiagen) and reverse transcribed (Superscript III, Invitrogen). Real-time PCR reactions were run using opsin-specific TaqMan primers and probes that spanned the exon–exon boundaries. The recently diverged *Rh2Aα* and *Rh2Aβ* opsin genes are genetically similar and produce visual pigments that differ in absorbance by only 10 nm [29]. As in previous studies, we quantified them together [25],[29],[30]. Reactions for all six opsin classes were run in parallel. An internal standard containing a tandem array of segments from each opsin gene was used to calculate the reaction efficiency within each run. The relative expression of each opsin as a fraction of total cone opsin expression was then calculated from the reaction efficiency and critical cycle number [28],[29]. Each reaction was run twice, and averages of both runs from all individuals of a species are reported.

We clustered species with quantitatively similar opsin gene expression profiles via hierarchical clustering. However, because multivariate methods such as hierarchical clustering are sensitive to factors with relatively larger values [59], we standardized the expression values of opsins expressed within single and double cones separately. To do this, we divided the relative expression of each opsin by the combined expression of all other opsins within the same cone type (*SWS1*, *SWS2B*, and *SWS2A* for single cones; *Rh2B*, *Rh2A*, and *LWS* for double cones; see below for a justification of these assignments). This normalization procedure provides equal weighting to opsins expressed within single cones versus those expressed within double cones. We then used the normalized opsin expression data to calculate Euclidean distances between species and clustered them using Ward's method. We identified the optimal number of clusters resulting from this analysis using the Connectivity, Dunn, and Silhouette cluster validation indexes [60]. Given a range of potential clusters, these indexes provide relative measures of support for each cluster size. Here, we tested for the presence of two to ten clusters. We implemented both hierarchical clustering and cluster validation statistics in the R package clValid [60].

### Calculating Single- and Double-Cone Sensitivity

We calculated the average single- and double-cone sensitivities of all taxa in order to better understand how changes in gene expression might influence overall retinal sensitivity. First, we assigned opsin genes to cone types. Based on MSP data from 19 Malawian cichlid species [44],[47],[61], nine Victorian cichlid species [25],[62], one Tanganyikan cichlid [46], and the riverine cichlid, *Oreochromis niloticus* [30], we have found that all cichlid single cones have a wavelength of maximum absorbance (λ_max_) that is less than 460 nm, and all cichlid double cones have a λ_max_ that is greater than 460 nm. Based on the λ_max_ of heterologously expressed opsins from *O. niloticus* [29] and *M. zebra* [44], this means that the *SWS1*, *SWS2B*, and *SWS2A* opsin genes are expressed in single cones, whereas *Rh2B*, *Rh2A*, and *LWS* are expressed in double cones.

To calculate average single- or double-cone sensitivities, peak spectral sensitivities for each opsin were weighted by the fraction of their expression in each cone type using the following equations: 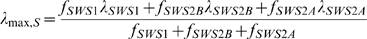
and
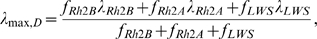
where *f_i_* is the relative expression and λ_i_ is the λ_max_ of one particular opsin [29],[30]. We used previously published λ_max_ values from heterologously expressed *O. niloticus* opsins (SWS1 = 360 nm, SWS2B = 425 nm, SWS2A = 456 nm, Rh2B = 472 nm, Rh2Aα+β = 523 nm [mean], and LWS = 560 nm) [29]. *O. niloticus* (Nile Tilapia) is considered an outgroup to both radiations [63]. As for the clustering of opsin expression values, we used the clValid [60] package to validate the number of single- and double-cone clusters (two to ten clusters) using the Dunn, Connectivity, and Silhouette measures of internal cluster support.

Finally, although opsin expression and visual pigment sensitivity are tightly correlated [25],[29],[30], these estimates of single- and double-cone sensitivity are not meant to suggest how colors are perceived (e.g., dichromacy vs. trichromacy). Rather, estimating single- and double-cone sensitivity allowed us to plot the data in a two-dimensional space to infer how changes in gene expression influence overall retinal sensitivity in a quantitative manner.

These single- and double-cone sensitivities were estimated based on two assumptions: (1) the visual pigment λ_max_ for each gene is the same for all species; and (2) the chromophore is A1 (11-*cis* retinal) for all species. We have not attempted to estimate individual λ_max_ values for each gene in each species for several reasons. First, we have not sequenced all the genes from all species. Second, we do not know the effects of all the sites, which vary across each of the opsins, and so would not be able to predict the exact λ_max_. However, based on the range of λ_max_ values that have been estimated from MSP of 30 different cichlid species from Lakes Malawi and Victoria, the variation in λ_max_ is relatively small: SWS1 371±8 nm, SWS2B 418±5 nm, SWS2A 455±5 nm, Rh2B 482±5 nm, Rh2A 528±6 nm, and LWS 565±9 nm (see Table 1 in [64]). Although there is larger variation in the SWS1 and LWS visual pigments, in agreement with our sequence diversity, this variation would have a negligible effect on the placement of species in their respective opsin expression clusters. Therefore, a reasonable approximation is to use the same λ_max_ for each gene in all species. Similarly, we have neglected any effects of chromophore switching from A1 to A2. Malawian cichlids utilize primarily A1 chromophore. However, Victorian cichlids do show some evidence of A2 usage. A complete chromophore switch causes small shifts for SWS1 (15 nm), SWS2B (7 nm), and SWS2A (10 nm), but larger shifts for Rh2B (19 nm), Rh2A (35 nm), and LWS (60 nm) based pigments [39]. It is more typical for the chromophore to be an A1/A2 mixture, which would decrease the size of these shifts. The net effect of A2 expression would be to push the double-cone estimates for Victorian cichlids to longer wavelengths. This would stretch the long-wavelength cluster, but would never cause Victorian species to shift into the shorter-wavelength clusters. Further studies are needed to quantify chromophore usage in wild-caught fish, as this could be important for actual visual sensitivities.

### Ecological Correlations within Lake Malawi

We used the phylogenetic comparative method [65] to test the hypothesis that opsin gene expression and the resulting single- and double-cone sensitivities differ among Lake Malawi cichlids with different foraging modes or macrohabitat preferences. Because of the lack of a resolved species-level phylogeny for this group, we used three different phylogenetic hypotheses for our analyses, a mitochondrial gene tree reconstructed from 1,247 bp of mtDNA, a generic tree illustrating the purported taxonomic relationships among the genera sampled, and a star tree in which the mbuna and non-mbuna clades were collapsed into polytomies (representing their rapid radiation from a common ancestor) (Figure S2A–S2C, Table S7). Additionally, we also performed a conservative nested ANOVA using only contrasts between species within each genus. A detailed discussion of how these phylogenetic hypotheses were generated and how uncertainties were dealt with is included in the supplementary materials (Text S2).

A phylogenetic ANOVA was implemented in the program PDSIMUL v2.0 [66]. Null distributions of *F*-statistics for ANOVA, corrected for phylogenetic nonindependence, were generated by simulation (*n* = 1,000) of relative opsin gene expression levels and estimated single- and double-cone λ_max_ values across the three trees listed above. These simulations followed an unbounded Brownian motion model of character evolution. All statistical analyses were performed using the stats functions and PHYLOGR [67] packages in the program R v2.6.2.

### Spectral Measurements

We measured the transmission properties of waters from Lakes Malawi and Victoria in the field. In Lake Malawi, the water attenuation coefficient as a function of wavelength was determined at two locations, Zimbawe Island, a rocky outcrop with a maximum depth of 40 m, and the southern side of Thumbi West Island, in a sheltered bay with a maximum depth of 15 m. A set of ten irradiance measurements were taken from a series of depths (0, 1, 3, 5, 7, 10, 15, and 20 m at Zimbawe and 0, 1, 3, 7, and 10 m at Thumbi West) using Subspec, a submersible Ocean Optics (USB 2000) spectrometer fitted with a 100-µm fiber and a cosine collector. These data were used to determine the slope (*k*, attenuation coefficient) and intercept (*b*) of a plot of ln(*I_d_*/*I*_0_) versus depth (*d*), where *I*_0_ is the initial, full-spectrum irradiance, and *I_d_* is the irradiance at depth. Transmission (T) at 2 m depth was then calculated using the equation T = *e* ^(k*d+*b*)^. Relative irradiance was then calculated by multiplying T by *I*_0_.

Victorian water measurements were taken at Makobe Island, a relatively clear location, Python Island, a turbid location, and Luanso Island, an extremely turbid location. Transmission was measured at a depth of 2 m for all three locations, using an AvaSpec 2048 212 spectrophotometer with a 10 m fiber cable (100 µm) and SpectraWin 4.16 software (Avantes). Measurements were taken in the shade, between 8h30 and 9h00 in the morning. Irradiance was then calculated by multiplying T by *I*_0_. The same *I*_0_ (from Zimbawe) was used for both Malawi and Victoria to remove any daily variation and focus only on differences in water properties.

### Calculating Relative Quantum Catch

We estimated the quantum catch (*Q*) that a visual pigment containing each opsin gene would have at each location in Lake Malawi and Victoria using the following equation:
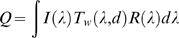
where *I*(λ) is the incident solar irradiance at the surface (measured at Zimbawe Rock), T*_w_*(λ,*d*) is the light transmission of the water to a depth (*d* = 2 m), and *R*(λ) is the photoreceptor absorption calculated using equations from Govardovskii et al. [68]. Because we were interested in the relative quantum catch each opsin gene would produce, we normalized the quantum catch for each visual pigment by the sum of the quantum catches from all visual pigments (this also removed intensity differences across geographic regions). Unpublished data suggest that ocular media are not limiting (e.g., species that express the UV opsin have UV-transmitting lenses). Therefore, the potential influence of ocular media was not included in this estimate.

### Ecological Correlations within Lake Victoria

To estimate the relative quantum catch that an SWS2B-based visual pigment would have at the location each taxon in Lake Victoria was collected, we first used Secchi disk readings (Table S4) to divide them into clear (>150 cm) or turbid locations (<150 cm). Because we did not have measurements of the light environment from all locations, we used the attenuation coefficient from Makobe to represent clear water and from Python to represent turbid water. The mean depth each taxon inhabits at the location where it was collected was used to calculate the transmission and relative irradiance. We then calculated the relative quantum catch that an SWS2B-based visual pigment would have in this light environment using the equation described above.

To test whether *SWS2B* expression was correlated with visual pigment quantum catch (Table S4), we used Felsenstein's independent contrasts method [65] as implemented in the PDAP v1.08 [69] module of Mesquite v1.11 [70]. Because of the rapid nature of the Victorian radiation (<100,000 y), we once again used a generic phylogeny for this analysis. To account for the presence of polytomies in this tree, we subtracted five degrees of freedom when calculating *p*-values for this analysis (Text S2).

### Opsin Sequence Diversity

We sequenced all seven cone opsin genes plus the rod opsin from five Lake Victoria taxa using previously published methods (Table S2). Genomic DNA was isolated from fin clips and amplified using opsin-specific PCR primers [28],[44],[61]. PCR products were gel or column purified and sequenced using PCR and internal primers. For all sequencing, we obtained at least 2× coverage and >95% of each gene's coding sequence.

Additional opsin sequences from previously published Lake Malawi and Victoria taxa were downloaded from GenBank (Table S2). Since the *Rh2Aα* and *Rh2B* gene sequences were missing for many of these taxa, we sequenced these genes for 18 taxa as well as any other missing or incomplete genes from genomic or cDNA stocks whenever possible (Table S2). Sequences were assembled and edited using Sequencher (v4.9, Genecodes Corp.). Consensus sequences were then aligned, and intronic regions were removed. Previously published alignments between each cichlid opsin and bovine rhodopsin were used to identify amino acid substitutions that fell in the putative transmembrane and retinal binding pocket regions [71]. Substitutions were then examined to determine whether they were between amino acids with different physical properties. These properties were nonpolar hydrophobic, polar uncharged, polar acidic, and polar basic. This approach was chosen because of previous work that suggests statistical tests of selection in opsins can be misleading [72]. To rule out the possibility that the changes we observed were due to differences in the mutation rates of different opsins, we used MEGA v4.0 [73] to calculate average pairwise *D_S_*, and π statistics for each opsin.

## Supporting Information

Figure S1 **Depiction of Malawian and Victorian opsin expression in a phylogenetic context.**
(0.34 MB PDF)

Figure S2 **Trees used for phylogenetically corrected statistical methods.**
(0.03 MB PDF)

Figure S3 **Synonymous substitution rates (*Ds*) and nucleotide diversity (π) of each opsin gene.**
(0.04 MB PDF)

Table S1 **Lake Malawi and Victoria species analyzed using real-time PCR.**
(0.06 MB PDF)

Table S2 **Accession numbers of all opsins included in this study.**
(0.06 MB PDF)

Table S3 **Summary of the phylogenetic ANOVA results.** These analyses compared relative opsin expression and single- and double-cone sensitivity to foraging mode and habitat among cichlid species from Lake Malawi using three different phylogenetic hypotheses (Figure S2).
(0.05 MB PDF)

Table S4 **Relative SWS2B-based visual pigment quantum catch, location, depth, and Secchi disc readings (cm) for Victorian taxa. **
(0.01 MB PDF)

Table S5 **Summary of amino acid variation in cichlid opsin genes from Lakes Malawi and Victoria.**
(0.05 MB PDF)

Table S6 **Substitutions between amino acids with different physical properties that were located in the transmembrane region.**
(0.06 MB PDF)

Table S7 **Accession numbers for mtDNA sequences used to generate phylogenies for the comparative methods in this study.**
(0.01 MB PDF)

Table S8 **Cluster validation statistics for the opsin expression and the single- and double-cone sensitivity clusters.**
(0.01 MB PDF)

Text S1 **Variation at known spectral tuning sites. **
(0.02 MB PDF)

Text S2 **Discussion and detailed description of the phylogenetic and comparative methods used in this study.**
(0.02 MB PDF)
