# Supplementary figures and images for: The Eyes Have It: Regulatory and Structural Changes Both Underlie Cichlid Visual Pigment Diversity
Source: PLoS Biol. 2009 Dec 22;7(12):e1000266. doi: 10.1371/journal.pbio.1000266 (PMC2790343; doi:10.1371/journal.pbio.1000266)

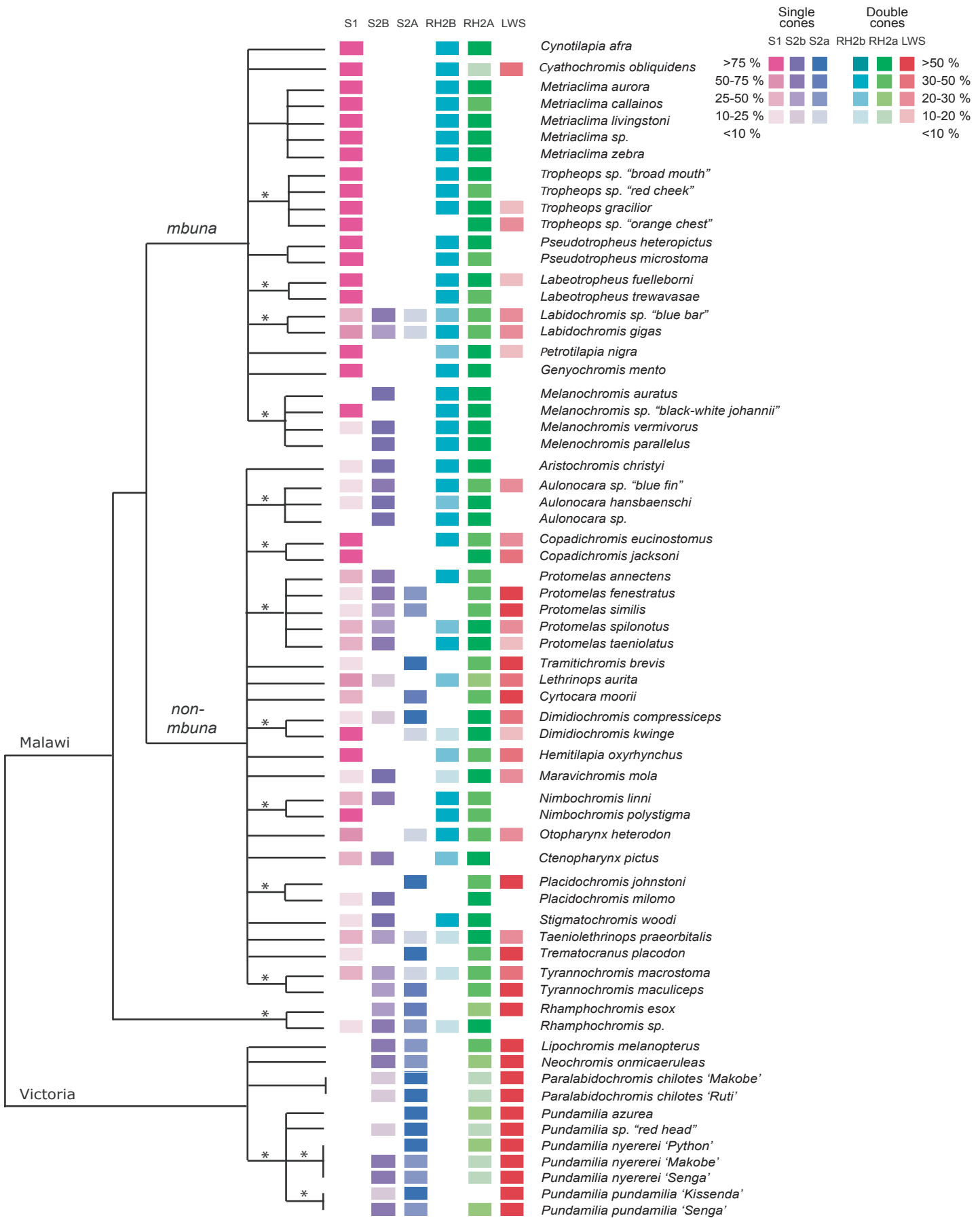

Supplement: Figure S1 — Depiction of Malawian and Victorian opsin expression in a phylogenetic context. (0.34 MB PDF) [file pbio.1000266.s001.pdf]

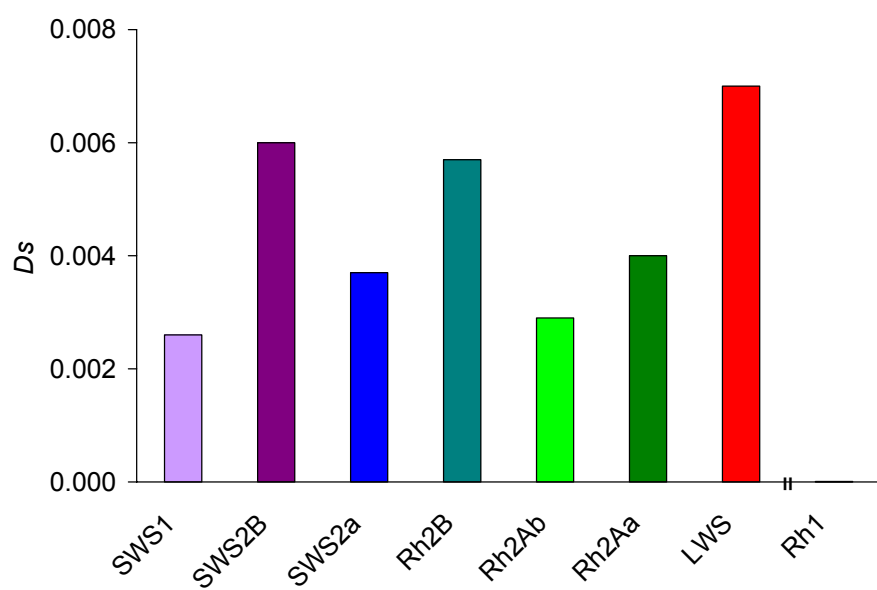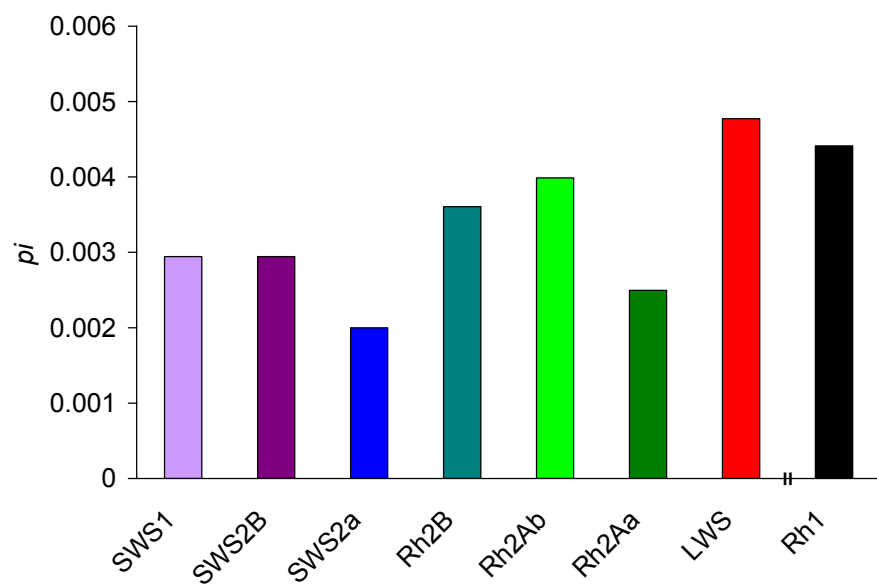

Supplement: Figure S3 — Synonymous substitution rates (Ds) and nucleotide diversity (π) of each opsin gene. (0.04 MB PDF) [file pbio.1000266.s003.pdf]
