# Supplementary material for: The Eyes Have It: Regulatory and Structural Changes Both Underlie Cichlid Visual Pigment Diversity
Source: PLoS Biol. 2009 Dec 22;7(12):e1000266. doi: 10.1371/journal.pbio.1000266 (PMC2790343; doi:10.1371/journal.pbio.1000266)
Supplement: Table S1 — Lake Malawi and Victoria species analyzed using real-time PCR. (0.06 MB PDF) [file pbio.1000266.s004.pdf]

Table S1. Lake Victoria and Malawi species analyzed using real-time PCR.

species	SWS1	SWS2B	SWS2A	Rh2B	Rh2A	LWS	single cone λmax (nm)	double cone λmax (nm)	cluster	location/clade	# individuals	foraging	habitat
**Victoria**													
Paralabidochromis chilotes	0.4	3.4	**23.6**	0.1	**15.0**	**57.4**	451	552	Long	Ruti	2	Benthos	Clear
"	0.5	2.5	**23.5**	0.1	**12.2**	**61.2**	451	554	Long	Makobe	1	Benthos	Clear
Pundamilia sp. "red head"	3.8	**23.3**	**11.5**	0.6	**13.2**	**47.7**	428	552	Long	Zue	2	Benthos	Turbid
Neochromis omnicaeruleas	1.6	**26.8**	**17.7**	0.2	**15.0**	**38.8**	435	550	Long	Ruti	2	Algae	Clear
Pundamilia azurea	1.0	2.0	**46.2**	0.1	**14.4**	**36.3**	453	550	Long	Ruti	2	Plankton, Algae	Clear
Lipochromis melanopterus	1.3	**37.6**	**13.1**	0.0	**14.9**	**33.1**	431	549	Long	Makobe	1	Predator (paedophage)	Clear
Pundamilia nyererei	1.0	2.2	**25.7**	0.1	**16.8**	**54.0**	450	552	Long	Python	5	Zooplankton, Omnivore	Very Turbid
"	2.9	**18.4**	**14.0**	0.2	**9.0**	**55.4**	432	555	Long	Makobe	2	"	Clear
"	0.7	**20.3**	**13.6**	0.1	**8.7**	**56.5**	436	555	Long	Senga	1	"	Clear
Pundamilia pundamilia	1.4	**22.8**	**14.3**	0.1	**12.4**	**49.0**	434	553	Long	Senga	4	Benthic insectivore, Omnivore	Clear
"	1.2	2.4	**12.6**	0.0	**5.3**	**78.5**	444	558	Long	Kissenda	1	"	Very Turbid
**Malawi**													
Cyathochromis obliquidens	**15.3**	0.6	0.2	**28.7**	**13.9**	**41.4**	363	524	Short	Mbuna	4	Loose Aufwuchs	Intermediate
Cynotilapia afra	**16.5**	0.1	0.0	**31.8**	**51.4**	0.1	361	505	Short	Mbuna	3	Zooplankton	Rock
Genyochromis mento	**15.9**	1.6	0.2	**33.4**	**48.6**	0.5	367	504	Short	Mbuna	2	Fish	Rock
Labeotropheus fuelleborni	**16.3**	0.6	0.6	**29.5**	**41.5**	**11.5**	365	511	Short	Mbuna	2	Algae	Rock
Labeotropheus trewavasae	**19.2**	0.5	0.1	**32.2**	**38.9**	**9.1**	362	508	Short	Mbuna	2	Algae	Rock
Labidochromis sp. "blue bar"	3.9	**7.7**	2.3	**20.0**	**42.3**	**23.7**	412	522	Middle	Mbuna	1	Loose Aufwuchs	Rock
Labidochromis gigas	**11.0**	**5.2**	2.5	**25.5**	**38.0**	**17.8**	391	516	Middle	Mbuna	3	Loose Aufwuchs	Rock
Melanochromis auratus	0.6	**21.8**	0.1	**33.7**	**39.4**	4.4	423	504	Middle	Mbuna	3	Algae	Rock
Melanochromis sp. "black-white johannii"	**22.2**	0.5	0.1	**27.4**	**46.1**	3.8	362	508	Short	Mbuna	3	Loose Aufwuchs	Intermediate
Melanochromis vermivorus	2.1	**18.4**	0.1	**33.9**	**42.8**	2.7	418	504	Middle	Mbuna	3	Loose Aufwuchs	Rock
Melanochromis parallelus	1.5	**21.4**	0.2	**36.5**	**38.9**	1.6	421	501	Middle	Mbuna	2	Loose Aufwuchs	Intermediate
Metriaclima aurora	**17.3**	1.0	0.2	**32.3**	**45.5**	3.6	365	505	Short	Mbuna	5	Algae	Intermediate
Metriaclima callainos	**17.3**	0.5	0.2	**36.1**	**40.8**	**5.1**	363	504	Short	Mbuna	2	Loose Aufwuchs	Rock
Metriaclima livingstonii	**15.6**	0.0	0.0	**33.9**	**46.4**	4.1	360	505	Short	Mbuna	1	Loose Aufwuchs	Rock
Metriaclima sp.	**12.9**	0.3	1.4	**33.8**	**44.5**	**7.2**	370	507	Short	Mbuna	1	Loose Aufwuchs	Rock
Metriaclima zebra	**13.4**	0.9	0.4	**30.3**	**52.4**	2.6	367	507	Short	Mbuna	3	Zooplankton	Rock
Petrotilapia nigra	**21.9**	0.4	0.6	**19.5**	**45.1**	**12.4**	364	517	Short	Mbuna	5	Loose Aufwuchs	Rock
Pseudotropheus heteropictus	**13.1**	0.7	0.2	**36.7**	**48.7**	0.7	365	503	Short	Mbuna	1	Zooplankton	Intermediate
Pseudotropheus microstoma	**28.0**	0.2	0.1	**32.6**	**34.3**	4.7	361	503	Short	Mbuna	2	Algae	Intermediate
Pseudotropheus tropheops red cheek	**18.0**	0.0	0.1	**37.3**	**40.9**	3.7	361	502	Short	Mbuna	1	Algae	Rock
Tropheops sp. "broad mouth"	**22.8**	0.1	0.0	**32.0**	**43.4**	1.7	360	504	Short	Mbuna	1	Algae	Intermediate
Tropheops gracillior	**18.8**	0.2	0.3	**28.6**	**43.0**	**9.1**	362	510	Short	Mbuna	4	Algae	Intermediate
Tropheops sp. "orange chest"	**15.0**	0.1	0.1	**8.0**	**56.5**	**20.3**	361	528	Short	Mbuna	1	Algae	Rock
Aristochromis christyi	4.9	**17.6**	0.8	**25.4**	**47.7**	3.5	412	509	Middle	Non-Mbuna	2	Fish	Intermediate
Aulonocara hansbaenschi	1.4	**8.5**	0.5	**19.0**	**55.2**	**15.3**	418	520	Middle	Non-Mbuna	2	Benthic Inverts	Intermediate
Aulonocara sp.	2.0	**18.1**	0.9	**35.7**	**40.8**	2.5	420	502	Middle	Non-Mbuna	1	Benthic Inverts	Intermediate
Aulonocara sp. "blue fin"	**6.4**	**18.3**	1.3	**22.9**	**29.8**	**21.3**	411	519	Middle	Non-Mbuna	4	Benthic Inverts	Intermediate
Copadichromis eucinostomus	**19.7**	1.1	0.2	**25.7**	**36.6**	**16.8**	364	515	Short	Non-Mbuna	4	Zooplankton	Sand
Copadichromis jacksoni	**15.0**	0.2	0.0	**6.7**	**50.3**	**27.7**	361	532	Short	Non-Mbuna	1	Zooplankton	Pelagic
Cyrtocara moorii	3.4	0.1	**6.9**	0.0	**36.9**	**52.8**	424	546	Long	Non-Mbuna	1	Benthic Inverts	Sand
Dimidiochromis compressiceps	1.2	1.3	**8.5**	0.0	**45.7**	**43.3**	442	542	Long	Non-Mbuna	1	Fish	Weed
Dimidiochromis kiwinge	**16.6**	1.4	2.3	**11.1**	**58.5**	**10.1**	375	522	Short	Non-Mbuna	1	Fish	Pelagic
Lethrinops aurita	**10.8**	3.5	0.3	**23.3**	**22.4**	**39.7**	377	527	Short	Non-Mbuna	4	Zooplankton	Sand
Maravichromis mola	2.7	**12.2**	0.4	**16.5**	**49.4**	**18.8**	414	522	Middle	Non-Mbuna	2	Benthic Inverts	Sand
Nimbochromis linni	**9.3**	**16.1**	0.8	**33.4**	**35.2**	**5.1**	403	503	Middle	Non-Mbuna	2	Fish	Intermediate
Nimbochromis polystigma	**19.0**	4.1	0.5	**33.1**	**31.5**	**11.8**	373	507	Short	Non-Mbuna	2	Fish	Intermediate
Otopharynx heterodon	**28.1**	**10.8**	**9.8**	**16.7**	**20.2**	**14.3**	394	518	Middle	Non-Mbuna	1	Loose Aufwuchs	Intermediate
Ctenopharynx pictus	**6.9**	**15.2**	0.6	**22.6**	**47.1**	**7.5**	406	513	Middle	Non-Mbuna	2	Loose Aufwuchs	Intermediate
Placidochromis johnstoni	1.2	1.1	**10.7**	0.1	**40.4**	**46.6**	445	544	Long	Non-Mbuna	1	Fish	Weed
Placidochromis milomo	1.7	**6.6**	0.2	**8.7**	**74.3**	**8.5**	413	523	Middle	Non-Mbuna	1	Benthic Inverts	Rock
Protomelas annectens	**11.0**	**27.7**	0.2	**30.5**	**29.5**	1.0	407	499	Middle	Non-Mbuna	1	Benthic Inverts	Sand
Protomelas fenestratus	2.1	**8.1**	**6.0**	**8.1**	**25.5**	**50.2**	428	541	Long	Non-Mbuna	1	Benthic Inverts	Intermediate
Protomelas similis	1.7	4.7	**5.3**	2.1	**26.6**	**59.5**	430	547	Long	Non-Mbuna	2	Algae	Weed
Protomelas spilonotus	**16.0**	**16.7**	**7.3**	**12.0**	**32.6**	**15.4**	405	523	Middle	Non-Mbuna	1	Zooplankton	Rock
Protomelas taeniolatus	**9.3**	**11.5**	0.3	**25.3**	**44.8**	**8.8**	397	512	Middle	Non-Mbuna	6	Loose Aufwuchs	Rock
Stigmatochromis woodi	**5.2**	**16.8**	0.2	**30.1**	**47.2**	0.5	410	505	Middle	Non-Mbuna	1	Fish	Pelagic
Taeniolatus praeorbitalis	**9.6**	6.0	3.9	**8.8**	**52.0**	**19.7**	399	528	Middle	Non-Mbuna	1	Benthic Inverts	Sand
Tramitichromis brevis	1.1	0.1	**7.0**	0.1	**31.9**	**59.9**	443	548	Long	Non-Mbuna	1	Benthic Inverts	Sand
Trematocranus placodon	0.9	0.2	**6.3**	0.1	**37.2**	**55.3**	444	546	Long	Non-Mbuna	2	Benthic Inverts	Intermediate
Tyrannochromis macrostoma	**7.5**	**9.3**	2.0	**9.7**	**35.5**	**36.0**	402	534	Middle	Non-Mbuna	2	Fish	Rock
Tyrannochromis maculiceps	1.2	4.5	**10.5**	0.9	**36.9**	**46.0**	440	544	Long	Non-Mbuna	1	Fish	Rock
Hemitilapia oxyrhynchus	**14.9**	0.2	0.1	**22.4**	**36.9**	**25.5**	361	522	Short	Non-Mbuna	2	Algae	Weed
Rhamphochromis esox	1.6	**5.3**	**12.1**	3.6	**20.7**	**56.7**	417	515	Middle	Pelagic-Haplochromine	1	Fish	Pelagic
Rhamphochromis sp.	1.4	2.9	1.5	**15.5**	**78.7**	0.0	439	547	Long	Pelagic-Haplochromine	1	Fish	Pelagic

Opsin expression is % of total. Opsins with greater than 5% relative expression are in bold.
Cluster refers to the sensitivity group that each taxon was assigned to.
