# Supplementary material for: The Eyes Have It: Regulatory and Structural Changes Both Underlie Cichlid Visual Pigment Diversity
Source: PLoS Biol. 2009 Dec 22;7(12):e1000266. doi: 10.1371/journal.pbio.1000266 (PMC2790343; doi:10.1371/journal.pbio.1000266)
Supplement: Table S2 — Accession numbers of all opsins included in this study. (0.06 MB PDF) [file pbio.1000266.s005.pdf]

Table S2. Accession numbers of all opsins included in this study.

	SWS1	SWS2b	SWS2a	RH2B	RH2Aα	RH2Aβ	LWS	RH1
**Lake Victoria**								
Pundamilia pundamilia	AY673729[1]	AY673709[1]	AY673719[1]<br>AY673760[1]<br>AY673761[1]	GQ422513	GQ422490	AY673699[1]<br>AY673753[1]<br>AY673754[1]	AY673689[1]<br>AY673748[1]<br>AY920395[1]	AY673739[1]<br>AY673767[1]
Pundamilia nyererei	AY673728[1]	AY6737088[1]	AY673718[1]<br>AY673762[1]<br>AY673763[1]	GQ422522	GQ422482	AY673698[1]<br>AY673755[1]<br>AY673756[1]	AY673688[1]<br>AY673749[1]<br>AY920394[1]	AY673738[1]<br>AY673768[1]
Pundamilia azurea	AY673730[1]	AY673710[1]	AY673720[1]<br>AY673764[1]	GQ422504,<br>GQ422506	GQ422487,<br>GQ422489	AY673700[1]<br>AY673757[1]	AY673690[1]<br>AY673750[1]	AY673740[1]<br>AY673769[1]
Pundamilia sp "red head"	AY673732[1]	AY673712[1]	AY673722[1]<br>AY673766[1]	GQ422505,<br>GQ422521	GQ422485,<br>GQ422488	AY673702[1]<br>AY673759[1]	AY673691[1]<br>AY673752[1]	AY673742[1]<br>AY673771[1]
Pundamilia luanso	AY673731[1]	AY673711[1]	AY673721[1]<br>AY673765[1]	GQ422510,<br>GQ422514	GQ422484,<br>GQ422496	AY673701[1]<br>AY673758[1]	AY673692[1]<br>AY673751[1]	AY673741[1]<br>AY673770[1]
Neochromis omnicaeruleus	AY673735	AY673715	AY673725	GQ422502,<br>GQ422503	GQ422481,<br>GQ422492	AY673705	AY673695	AY673745
Neochromis greenwoodi	AY673734	AY673714	AY673724	GQ422512	GQ422495	AY673704	AY673694	AY673744
Lipochromis melanopterus	AY673733	AY673713	AY673723	GQ422507,<br>GQ422509	GQ422486,<br>GQ422491	AY673703	AY673693	AY673743
Paralabidochromis chilotes	AY673736	AY673716	AY673726	GQ422511	GQ422493	AY673706	AY673696	AY673746
Paralabidochromis cyanus	AY673737	AY673717	AY673727	GQ422508	GQ422494	AY673707	AY673697	AY673747
**Lake Malawi**								
Aulonocara hueseri	AY775100[2]	AY775083[2]	AY775074[2]	GQ422516	GQ422480	AY775090[2]	AY780517[2]	AY775112[2]
Aulonocara baenschi	GQ422525	GQ422528	GQ422527	GQ422520	GQ422499	GQ422500	GQ452104	GQ422474
Cynotilapia afra	AY775104[2]	AY775088[2]	AY775079[2]	GQ422517	GQ422497	AY775094[2]	AY780521[2]	AY775118[2]
Labeotropheus fuelleborni	AF191223[3]	AF247119[4]	AF247115[4]			AF247123[4]	AF247127[4]	AY775113[2]
Labidochromis chisumulae	AY775098[2]	AY775064[2]	AY775081[2]	GQ422518	GQ422478	AY775069[2]	AY780515[2]	AY775120[2]
Melanochromis auratus	AY775101[2]	AY775084[2]	AY775076[2]	GQ422515	GQ422483	AY775091[2]	AY780518[2]	AY775115[2]
Melanochromis vermivorus	DQ088643[5]	DQ088640[5]	DQ088637[5]	DQ088646[5]	DQ088631[5]	DQ088634[5]	DQ088628[5]	GQ422472
Metriaclima zebra	AF191219[3]<br>AF191222[3]	AF317674[3]<br>AF247118[4]	AF247114[4]	DQ088652[5]	DQ088651[5]	AF247122[4]	AF247126[4]	AY775114[2]
Pseudotropheus acei	DQ088642[5]	DQ088639[5]	DQ088636[5]	DQ088645[5]	DQ088630[5]	DQ088633[5]	DQ088627[5]	GQ422475
Copadichromis borleyi	AY775106[2]	AY775065[2]	AY775061[2]			AY775071[2]	AY780514[2]	AY775121[2]
Dimidiochromis compressiceps	AF191220[3]	AF247117[4]	AF247113[4]			AF247121[4]	AF247125[4]	AY775059[2]
Lethrinops parvidens	AY775102[2]	AY775087[2]	AY775077[2]		GQ422477	AY775092[2]	AY780519[2]	AY775116[2]
Mylochromis lateristriga	AY775105[2]	AY775085[2]	AY775075[2]	GQ452103	GQ422476	AY775095[2]	AY780522[2]	AY775119[2]
Stigmatochromis modestus	AY775107[2]	AY775066[2]	AY775080[2]			AY775070[2]	AY780523[2]	AY775122[2]
Tramitichromis intermedius	DQ088644[5],<br>*GQ422524*	DQ088641[5],<br>GQ422529	DQ088638[5],<br>GQ422526	DQ088647[5],<br>GQ422523	DQ088632[5],<br>GQ422498	DQ088635[5],<br>GQ422501	GQ452105	GQ422473
Tyrannochromis maculatus	AY775103[2]	AY775086[2]	AY775078[2]	GQ422519	GQ422479	AY775093[2]	AY780520[2]	AY775117[2]

[1]Carleton et al. 2005. Mol Ecol 14: 4341–4353.

[2]Spady et al. 2005. Mol Biol Evol 22: 1412–1422.

[3]Carleton et al. 2000. Vision Res 40: 879-890.

[4]Carleton and Kocher 2001. Mol Biol Evol 18: 1540–1550.

[5]Parry et al. 2005. Curr Biol 15: 1–6.
