# Supplementary material for: The Eyes Have It: Regulatory and Structural Changes Both Underlie Cichlid Visual Pigment Diversity
Source: PLoS Biol. 2009 Dec 22;7(12):e1000266. doi: 10.1371/journal.pbio.1000266 (PMC2790343; doi:10.1371/journal.pbio.1000266)
Supplement: Table S3 — Summary of the phylogenetic ANOVA results. These analyses compared relative opsin expression and single- and double-cone sensitivity to foraging mode and habitat among cichlid species from Lake Malawi using three different phylogenetic hypotheses (Figure S2). (0.05 MB PDF) [file pbio.1000266.s006.pdf]

Table S3. Results from a phylogenetic ANOVA comparing relative opsin expression and single and double cone sensitivity to foraging mode and habitat among cichlid species from Lake Malawi using three different phylogenetic hypotheses (Fig. S2).

Mitochondria	Habitat		Foraging	
	$F_{4,45}$	P	$F_{4,45}$	P
SWS1	2.229	0.456	7.647	**0.007**
SWS2b	0.332	0.977	1.234	0.598
SWS2a	1.468	0.656	1.957	0.367
RH2b	4.294	0.066	3.323	0.141
RH2a	3.127	0.214	0.755	0.810
LWS	4.345	0.061	1.147	0.636
Single Cones	2.179	0.493	9.065	**0.002**
Double Cones	4.084	0.085	2.067	0.363

Generic	Habitat		Foraging	
	$F_{4,45}$	P	$F_{4,45}$	P
SWS1	2.229	0.344	7.647	0.034
SWS2b	0.332	0.951	1.234	0.761
SWS2a	1.468	0.513	1.957	0.583
RH2b	4.294	0.064	3.323	0.310
RH2a	3.127	0.162	0.755	0.882
LWS	4.345	0.056	1.147	0.789
Single Cones	2.179	0.343	9.065	**0.012**
Double Cones	4.084	0.077	2.067	0.531

Star	Habitat		Foraging	
	$F_{4,45}$	P	$F_{4,45}$	P
SWS1	2.229	0.497	7.647	0.037
SWS2b	0.332	0.976	1.234	0.760
SWS2a	1.468	0.713	1.957	0.566
RH2b	4.294	0.109	3.323	0.364
RH2a	3.127	0.305	0.755	0.875
LWS	4.345	0.099	1.147	0.763
Single Cones	2.179	0.506	9.065	**0.010**
Double Cones	4.084	0.104	2.067	0.569

Values in bold are significant following Bonferroni correction for 8 hypothesis tests at an experiment-wise error rate of 10% (α = 0.10/8 = 0.0125)[Quinn and Keough 2002].
