# Supplementary material for: The Eyes Have It: Regulatory and Structural Changes Both Underlie Cichlid Visual Pigment Diversity
Source: PLoS Biol. 2009 Dec 22;7(12):e1000266. doi: 10.1371/journal.pbio.1000266 (PMC2790343; doi:10.1371/journal.pbio.1000266)
Supplement: Table S4 — Relative SWS2B-based visual pigment quantum catch, location, depth, and Secchi disc readings (cm) for Victorian taxa. (0.01 MB PDF) [file pbio.1000266.s007.pdf]

Table S4. Relative SWS2B quantum catch, location, depth and secchi disc readings for Victoria taxa.

	Location	Depth (m)	Secchi (cm)	SWS2B quantum catch (%)	SWS2B expression (%)
Paralabidochromis chilotes	Ruti	10	223	0.09	3.43
"	Makobe	2	225	4.29	2.48
Pundamilia sp. "red head"	Zue	2	150	4.29	23.29
Neochromis omnicaeruleas	Ruti	4	223	1.38	26.75
Pundamilia azurea	Ruti	12	223	0.05	2.04
Lipochromis melanopterus	Makobe	1	225	7.58	37.59
Pundamilia nyererei	Python	4	98	0.04	2.25
"	Makobe	6	225	0.49	18.39
"	Senga	6	200	0.49	20.34
Pundamilia pundamilia	Senga	2	200	4.29	22.79
"	Kissenda	2	78	0.37	2.42
