# Supplementary material for: The Eyes Have It: Regulatory and Structural Changes Both Underlie Cichlid Visual Pigment Diversity
Source: PLoS Biol. 2009 Dec 22;7(12):e1000266. doi: 10.1371/journal.pbio.1000266 (PMC2790343; doi:10.1371/journal.pbio.1000266)
Supplement: Table S5 — Summary of amino acid variation in cichlid opsin genes from Lakes Malawi and Victoria. (0.05 MB PDF) [file pbio.1000266.s008.pdf]

Table S5. Summary of amino acid variation in cichlid opsin genes from Lakes Malawi and Victoria.

	SWS1	SWS2B	SWS2A	Rh2B	Rh2Aα	Rh2Aβ	LWS	Rh1
nucleotide substitutions	26	16	15	16	22	32	24	22
indels			(+1codon)				(-1codon)	
variable aa sites - entire protein	21	9	6	10	10	22	15	15
non-synonymous aa substitutions - entire protein	9	2	2	3	1	3	9	7
variable aa sites - trans-membrane regions	16	5	3	7	6	16	12	13
non-synonymous aa substitutions - trans-membrane regions	7	1	1	1	1	3	8	7
variable aa sites - retinal binding pocket	5	3	1	2	1	3	5	3
non-synonymous aa substitutions - retinal binding pocket	3	1	0	1	0	0	5	3
substitutions unique to Lake Victoria	3	1	1	1	1	0	6	9
fixed differences between lakes	1	0	1	1	1	0	1	1

Non-synonymous substitutions are between amino acids (aa) with different chemical properties (nonpolar hydrophobic, polar uncharged, polar basic, polar acidic).
