# Supplementary material for: The Eyes Have It: Regulatory and Structural Changes Both Underlie Cichlid Visual Pigment Diversity
Source: PLoS Biol. 2009 Dec 22;7(12):e1000266. doi: 10.1371/journal.pbio.1000266 (PMC2790343; doi:10.1371/journal.pbio.1000266)
Supplement: Table S6 — Substitutions between amino acids with different physical properties that were located in the transmembrane region. (0.06 MB PDF) [file pbio.1000266.s009.pdf]

Table S6. Substitutions between amino acids with different physical properties that were located in the transmembrane region. Retinal binding pocket sites are shaded grey. Sites that have been previously demonstrated to tune opsin spectral sensitivity and that vary among cichlids are marked at the bottom of the table. In addition, two sites that correspond with measured differences in cichlid sensitivity are highlighted. Numbers correspond to bovine rhodopsin. Abbreviations for the amino acid physical properties are as follows nonpolar hydrophobic (NPH), polar uncharged (PU), polar acidic (PA), and polar basic (PB). The top middle and bottom panels correspond to Malawi rock, Malawi sand, and Victoria groups.

	SWS1							SWS2B	SWS2A	RH2B	RH2Ab			RH2Aa	LWS								Rh1						
	37	114	160	166	204	217	248	269	39	124	107	151	218	151	40	123	155	164	203	217	261	262	42	158	163	166	213	298	299
Aulonocara hueseri	Y	S	T	G	T	S	K	A	A	S	A	T	V	T	S/A	T/A	A	A	Y	A	Y	C	A	A	A	S	T	A	A
Aulonocara baenschi	F	G	A	G	I	S	K	A	A	S	S	T	T/I	A	S	T	A	A	Y	A	F/Y	C	A	A	A	S	T	A	A
Cynotilapia afra	F	S	T	G	T	S	K	A	A	S	S	A	V	A	S/A	T	A	A	Y	A	F	C	A	A	A	S	T	A	A
Labeotropheus fuelleborni	F	S	T	G	T	S	K	A	A		S	A	I		A	A	A	A	Y	A	Y	C	A	A	A	S	T	A	A
Labidochromis chisumulae	F/Y	S	T	G	T	S	K	A	T	S	P	A	I	A	A	A	A	A	Y	A	Y	C	A	A	A	S	T	A	A
Melanochromis auratus	Y	S	T	G	T	F	K	A	A	S	S	A	T	A	A	A	A	S	Y	A	Y	C	A	A	A	S	T	A	A
Melanochromis vermivorus	F	S	T	G	T	S	K	A	A	S	S	A	V	A	A	A	A	S	Y	A	Y	C	A	A	A	A	T	S	S
Metriaclima zebra	F	S[1]	T	G	T	S	K	A	T	S	S	A	I	A	A	A	A	A	Y	A	Y	C	A	A	A	S	T	A	A
Pseudotropheus acei	F	A[2]	A	A	I	S	K	A	T	S	S	A	V/I	A	A	A	A	S	Y	A	Y	C	A	A	A	A	T	S	S
Copadichromis borleyi	Y	S	T	G	T	S	K	A	A		S	T	I		A	A	A	A	Y	A	Y	C	A	A	A	A	I	S	S
Dimidiochromis compressiceps	F	S	T	G	T	S	K	T	A		S	A	I		A	A	A	A	Y	A	Y	C[3]	A	A	A	A	I	S	S
Lethrinops parvidens	F	S/T	T/A	G	T/I	S	K	A	A		P	A	I	A	S	A	A	S	Y	A	Y	C	A	A	A	A	I	S	S
Mylochromis lateristriga	F	S	T	G	T	S	K	A	A	A	S	T	I	A	A	A	A	A	Y	A	Y	C	A	A	A	A	T	S	S
Stigmatochromis modestus	F	S	T	G	T	S	E	A	A		A	T	T		S	T	A	A	Y	A	Y	C	A	A	A	A	T	A	A
Tramitichromis intermedius	F	S	T	G	T	S	K	T	A	S	P	A	I	A	A	A	A	A	Y	A	Y	C[3]	A	A	A	S	T	A	A
Tyrannochromis maculatus	F	S	T	G	T	S	K	A	A	A	S	T	I	A	A	A	A	A	Y	A	Y	C	A	A	A	A	I	A	A
Lipochromis melanopterus	F	A	A	G	I	S	K	T	A	S	P	A	I	A	A	A	A	A	Y	A	Y	C	A	A/G	A	S	?	A	A
Neochromis greenwoodi	F	A	A	G	I	S	K		A	S	P	A	I	A	A	A	A	S	Y	A	Y	I	C	G	G	S	L	A	A
Neochromis omnicaeruleus	F	A	A	S	I	S	K	T	A	S	P	A	I	A	A	A	A	A	Y	A	Y	C	C	G	G	S	L	A	A
Paralabidochromis chilotes	F	A	A	G	I	S	K	T	A	S	P	A	I	A	A	A	A	A	F	T	Y	I	C	G	G	S	L	A	A
Paralabidochromis cyaneus	F	A	A	G	I	S	K	A	A	S	P	A	I	A	A	A	G	S	Y	A	Y	C	A	A	A	S	L	A	A
Pundamilia azurea	F	A	A	S	I	S	K	T	A	S	P	A	I	A	A	A	A	A	Y	T	Y	I[4]	C	G	G	S	L	A	A
Pundamilia luanso	F	A	A	S	I	S	K	T	A	S	P	A	I	A	A	A	A	A	F	T	Y	I[4]	C	G	G	S	L	A	A
Pundamilia nyererei	F	A	A	G	I	S	K	T	A	S	P	A	I	A	A	A	A	A	Y	A	Y	C[3]	C	G	G	S	L	A	A
Pundamilia nyererei									A		P	A	I		A	A	A	A	F	T	Y	I[4]	C	G	G	S	L	A	A
Pundamilia nyererei									A		P	A	I		A	A	A	A	Y	A	Y	C							
Pundamilia pundamilia	F	A	A	G	I	S	K	T	A	S	P	A	I	A	A	A	A	A	F	T	Y	I[4]	C	G	G	S	L	A	A
Pundamilia redhead	F	A	A	G	I	S	K	T	A	S	P	A	I	A	A	A	A	A	Y	A	Y	C[3]	A	A	A	S	L	A	A
	F-NPH	S-PU	T-PU	GS-PU	T-PU	S-PU	K-PB	A-NPH	A-NPH	S-PU	S-PU	A-NPH	V,I-NPH	T-PU	S-PU	T-PU	A-NPH	A-NPH	Y-PU	A-NPH	Y-PU	I-NPH	A-NPH	A-NPH	A-NPH	S-PU	T-PU	A-NPH	A-NPH
	Y-PU	A-NPH	A-NPH	A-NPH	I-NPH	F-NPH	E-PA	T-PU	T-PU	A-NPH	AP-NPH	T-PU	T-PU	A-NPH	A-NPH	A-NPH	G-PU	S-PU	F-NPH	T-PU	F-NPH	C-PU	C-PU	G-PU	G-PU	A-NPH	I,L-NPH	S-PU	S-PU
	TM 1	TM3	TM4		TM5		TM 6	TM6	TM1	TM3	TM3	TM4	TM5	TM4	TM I	TM3	TM4		TM5		TM6		TM1		TM4		TM5	TM7	
		A-G=10 nm[5]						T-A=11 nm[6]										S-A=7 nm[7]			F-Y=10 nm[7]								

[1]*M. zebra* SWS1 has a λmax of 368 nm (Carleton et al. 2000. Vision Res 40: 879-890).

[2]*P. acei* SWS1 has a λmax 378 nm (Parry et al. 2005. Curr Biol 15: 1–6).

[3]These taxa have LWS λmax values that range from 567-569 nm (Carleton et al. 2005. Mol Ecol 14: 4341–4353; Parry et al. 2005. Curr Biol 15: 1–6).

[4]These taxa have LWS λmax values that range from 562-565 nm (Carleton et al. 2005. Mol Ecol 14: 4341–4353; Parry et al. 2005. Curr Biol 15: 1–6).

[5]Shi et al. 2001. Proc Natl Acad Sci USA 98: 11731–11736.

[6]Cowing et al. 2002. Biochemistry 41: 6019-6025.

[7]Asenjo et al. 1994. Neuron 12: 1131-1138.
