# Supplementary material for: The Eyes Have It: Regulatory and Structural Changes Both Underlie Cichlid Visual Pigment Diversity
Source: PLoS Biol. 2009 Dec 22;7(12):e1000266. doi: 10.1371/journal.pbio.1000266 (PMC2790343; doi:10.1371/journal.pbio.1000266)
Supplement: Table S7 — Accession numbers for mtDNA sequences used to generate phylogenies for the comparative methods in this study. (0.01 MB PDF) [file pbio.1000266.s010.pdf]

Table S7. Accession numbers for mtDNA sequences used to generate phylogenies for the comparative methods in this study.

species	ND2	Control
Aristochromis christyi	EF585282[1]	EF647535
Aulonocara baenschi	GQ422572	GQ422532
Aulonocara sp.	GQ422580	GQ422533
Aulonocara sp. "blue fin"	GQ422566	GQ422531
Copadichromis eucinostomus	EF585268	GQ422530
Copadichromis jacksoni	GQ422593	EF647580
Ctenopharynx pictus	GQ422587	GQ422547
Cyathochromis obliquidens	GQ422579	U90759[3]
Cynotilapia afra	EF585264[1]	AY911740
Cyrtocara moorii	AY930089[2]	U01105[3]
Dimidiochromis compressiceps	EF585267[1]	EF647532
Dimidiochromis kiwinge	AF305322	AJ291408[5]
Genyochromis mento	AF305297	U90779[3]
Hemitilapia oxyrhynchus	EF585277[1]	GQ422534
Labeotropheus fuelleborni	EF585259[1]	U90774[3]
Labeotropheus trewavasae	GQ422577	GQ422535
Labidochromis gigas	EF585276[1]	GQ422538
Labidochromis sp. "blue bar"	GQ422573	GQ422537
Lethrinops aurita	GQ422586	GQ422539
Maravichromis mola	EF585274[1]	GQ422540
Melanochromis auratus	AY930069[2]	U01107[3]
Melanochromis sp. "black-white johanni"	GQ422574	U01942[3]
Melanochromis vermivorus	EF585270[1]	GQ422541
Melenochromis parallelus	GQ422592	U01953[3]
Metriaclima aurora	GQ422569	GQ422542
Metriaclima callainos	GQ422570	AF2136204[4]
Metriaclima livingstonii	GQ422582	GQ422543
Metriaclima sp.	GQ422581	GQ422544
Metriaclima zebra	DQ093114	AY930025[2]
Nimbochromis linni	EF585279[1]	AY913941
Nimbochromis polystigma	EF585262[1]	AJ291407[5]
Otopharynx heterodon	EF585278[1]	GQ422546
Petrotilapia nigra	GQ422567	GQ422548
Placidochromis johnstoni	EF585269[1]	GQ422549
Placidochromis milomo	GQ422590	GQ422550
Protomelas annectens	GQ422575	AJ291414[5]
Protomelas fenestratus	AF305301	GQ422551
Protomelas similis	GQ422585	GQ422552
Protomelas spilonotus	EF585253	GQ422553
Protomelas taeniolatus	AF305302	EF647546
Pseudotropheus heteropictus	GQ422584	GQ422554
Pseudotropheus microstoma	EF585258[1]	GQ422555
Rhamphochromis esox	AF305252	AF298913
Rhamphochromis sp.	GQ422591	GQ422556
Stigmatochromis woodi	AF213626	GQ422557
Taeniolatus praeorbitalis	GQ422576	GQ422558
Tramitichromis brevis	AF305320	GQ422559
Trematocranus placodon	EF585261[1]	GQ422560
Tropheops gracillior	EF585260[1]	GQ422562
Tropheops sp. "broad mouth"	GQ422589	GQ422561
Tropheops sp. "orange chest"	GQ422583	GQ422563
Tropheops sp. "red cheek"	GQ422568	GQ422564
Tyrannochromis macrostoma	EF585257[1]	EF647537
Tyrannochromis maculiceps	GQ422571	GQ422565

[1]Hulsey et al. 2007. Proc R Soc B 274: 1867–1875.
[2]Salzburger et al. 2005. BMC Evol Biol 5: 17.
[3]Parker and Kornfield 1997. J Mol Evol 45: 70–83.
[4]Nagl et al. 2000 Proc R Soc B 267: 1049–1061.
[5]Meyer et al. 1990. Nature 347: 550–553.
