# Supplementary material for: The Eyes Have It: Regulatory and Structural Changes Both Underlie Cichlid Visual Pigment Diversity
Source: PLoS Biol. 2009 Dec 22;7(12):e1000266. doi: 10.1371/journal.pbio.1000266 (PMC2790343; doi:10.1371/journal.pbio.1000266)
Supplement: Table S8 — Cluster validation statistics for the opsin expression and the single- and double-cone sensitivity clusters. (0.01 MB PDF) [file pbio.1000266.s011.pdf]

Table S8. Three distance-based cluster validation statistics for cichlid opsin expression and photoreceptor sensitivities.

		Cluster sizes								
Variables	Index	2	3	4	5	6	7	8	9	10
Opsins	Connectivity	**2.698**	3.270	7.174	13.794	26.208	33.822	42.737	44.785	51.397
	Dunn	0.196	**0.311**	0.242	0.309	0.128	0.128	0.128	0.152	0.184
	Silhouette	0.460	**0.576**	0.566	0.511	0.443	0.413	0.384	0.392	0.407
Single and double cone sensitivity	Connectivity	**0.590**	**0.590**	7.193	15.540	24.122	29.604	35.075	39.434	42.214
	Dunn	0.242	**0.449**	0.153	0.199	0.116	0.140	0.140	0.140	0.184
	Silhouette	0.572	**0.674**	0.620	0.558	0.486	0.496	0.509	0.515	0.542

The optimal number of cluster(s) for each statistic is highlighted in bold.
