# Supplementary material for: The Eyes Have It: Regulatory and Structural Changes Both Underlie Cichlid Visual Pigment Diversity
Source: PLoS Biol. 2009 Dec 22;7(12):e1000266. doi: 10.1371/journal.pbio.1000266 (PMC2790343; doi:10.1371/journal.pbio.1000266)
Supplement: Figure S2 — Trees used for phylogenetically corrected statistical methods. (0.03 MB PDF) [file pbio.1000266.s002.pdf]

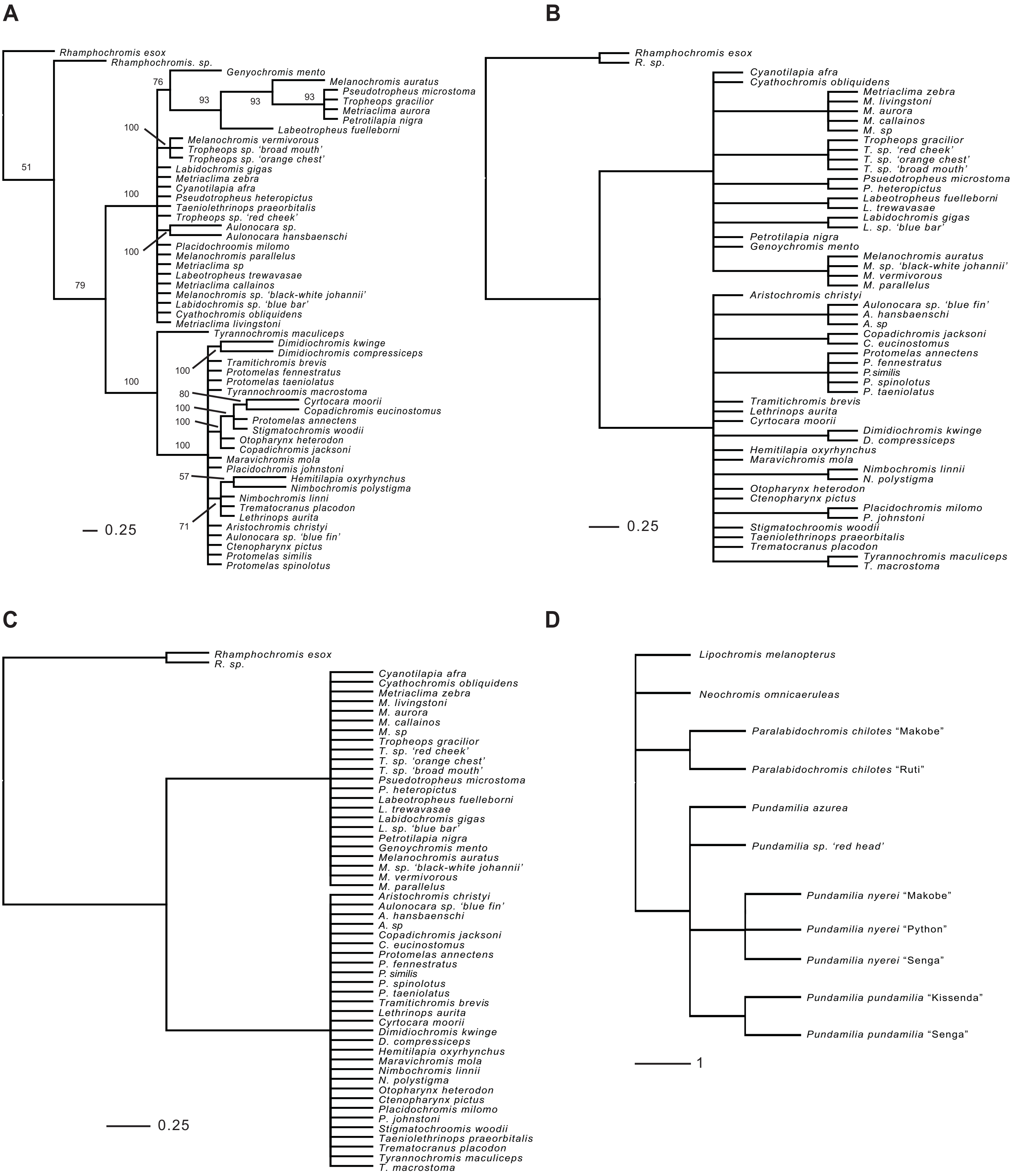

A
Rhamphochromis esox
Rhamphochromis. sp.
Genyochromis mento
Melanochromis auratus
Pseudotropheus microstoma
Tropheops gracilior
Metriaclima aurora
Petrotilapia nigra
Labeotropheus fuelleborni
Melanochromis vermivorous
Tropheops sp. 'broad mouth'
Tropheops sp. 'orange chest'
Labidochromis gigas
Metriaclima zebra
Cyanotilapia afra
Pseudotropheus heteropictus
Taeniolethrinops praeorbitalis
Tropheops sp. 'red cheek'
Aulonocara sp.
Aulonocara hansbaenschi
Placidochroomis milomo
Melanochromis parallelus
Metriaclima sp
Labeotropheus trewavasae
Metriaclima callainos
Melanochromis sp. 'black-white johannii'
Labidochromis sp. 'blue bar'
Cyathochromis obliquidens
Metriaclima livingstoni
Tyrannochromis maculiceps
Dimidiochromis kwinge
Dimidiochromis compressiceps
Tramitichromis brevis
Protomelas fennestratus
Protomelas taeniolatus
Tyrannochroomis macrostoma
Cyrtocara moorii
Copadichromis eucinostomus
Protomelas annectens
Stigmatochromis woodii
Otopharynx heterodon
Copadichromis jacksoni
Maravichromis mola
Placidochromis johnstoni
Hemitilapia oxyrhynchus
Nimbochromis polystigma
Nimbochromis linni
Trematocranus placodon
Lethrinops aurita
Aristochromis christyi
Aulonocara sp. 'blue fin'
Ctenopharynx pictus
Protomelas similis
Protomelas spinolotus
51
79
76
93
93
93
100
100
100
100
100
80
100
100
100
57
71
0.25
B
Rhamphochromis esox
R. sp.
Cyanotilapia afra
Cyathochromis obliquidens
Metriaclima zebra
M. livingstoni
M. aurora
M. callainos
M. sp
Tropheops gracilior
T. sp. 'red cheek'
T. sp. 'orange chest'
T. sp. 'broad mouth'
Psuedotropheus microstoma
P. heteropictus
Labeotropheus fuelleborni
L. trewavasae
Labidochromis gigas
L. sp. 'blue bar'
Petrotilapia nigra
Genoychromis mento
Melanochromis auratus
M. sp. 'black-white johannii'
M. vermivorous
M. parallelus
Aristochromis christyi
Aulonocara sp. 'blue fin'
A. hansbaenschi
A. sp
Copadichromis jacksoni
C. eucinostomus
Protomelas annectens
P. fennestratus
P.similis
P. spinolotus
P. taeniolatus
Tramitichromis brevis
Lethrinops aurita
Cyrtocara moorii
Dimidiochromis kwinge
D. compressiceps
Hemitilapia oxyrhynchus
Maravichromis mola
Nimbochromis linnii
N. polystigma
Otopharynx heterodon
Ctenopharynx pictus
Placidochromis milomo
P. johnstoni
Stigmatochroomis woodii
Taeniolethrinops praeorbitalis
Trematocranus placodon
Tyrannochromis maculiceps
T. macrostoma
0.25
C
Rhamphochromis esox
R. sp.
Cyanotilapia afra
Cyathochromis obliquidens
Metriaclima zebra
M. livingstoni
M. aurora
M. callainos
M. sp
Tropheops gracilior
T. sp. 'red cheek'
T. sp. 'orange chest'
T. sp. 'broad mouth'
Psuedotropheus microstoma
P. heteropictus
Labeotropheus fuelleborni
L. trewavasae
Labidochromis gigas
L. sp. 'blue bar'
Petrotilapia nigra
Genoychromis mento
Melanochromis auratus
M. sp. 'black-white johannii'
M. vermivorous
M. parallelus
Aristochromis christyi
Aulonocara sp. 'blue fin'
A. hansbaenschi
A. sp
Copadichromis jacksoni
C. eucinostomus
Protomelas annectens
P. fennestratus
P. similis
P. spinolotus
P. taeniolatus
Tramitichromis brevis
Lethrinops aurita
Cyrtocara moorii
Dimidiochromis kwinge
D. compressiceps
Hemitilapia oxyrhynchus
Maravichromis mola
Nimbochromis linnii
N. polystigma
Otopharynx heterodon
Ctenopharynx pictus
Placidochromis milomo
P. johnstoni
Stigmatochroomis woodii
Taeniolethrinops praeorbitalis
Trematocranus placodon
Tyrannochromis maculiceps
T. macrostoma
0.25
D
Lipochromis melanopterus
Neochromis omnicaeruleas
Paralabidochromis chilotes "Makobe"
Paralabidochromis chilotes "Ruti"
Pundamilia azurea
Pundamilia sp. 'red head'
Pundamilia nyerei "Makobe"
Pundamilia nyerei "Python"
Pundamilia nyerei "Senga"
Pundamilia pundamilia "Kissenda"
Pundamilia pundamilia "Senga"
1
