# Supplementary material for: The Eyes Have It: Regulatory and Structural Changes Both Underlie Cichlid Visual Pigment Diversity
Source: PLoS Biol. 2009 Dec 22;7(12):e1000266. doi: 10.1371/journal.pbio.1000266 (PMC2790343; doi:10.1371/journal.pbio.1000266)
Supplement: Text S2 — Discussion and detailed description of the phylogenetic and comparative methods used in this study. (0.02 MB PDF) [file pbio.1000266.s013.pdf]

Text S2. Phylogenetic and Comparative Methods

Mitochondrial gene tree reconstruction. We utilized 1,247 bp of mtDNA corresponding to 934 bp of the protein-coding gene ND2 and 313 bp of the mitochondrial control region to generate a mitochondrial gene tree for subsequent phylogenetically-controlled statistical analyses. Previously published sequences were available for approximately half the taxa sampled; the remaining sequences were obtained via PCR and direct sequencing following standard protocols [S1, S2] (Table S7). Multiple sequence alignment for the final sequence set was performed using the L-INS-i strategy of the program MAFFT [S3] and then edited manually.

We used MrBayes v3.1.2 [S4] to generate a phylogeny of all Lake Malawi species sampled. We specified the GTR+I+G model of nucleotide substitution for these analyses following likelihood ratio tests in the program MrModeltest v2.2 [S5]. We treated all model parameters as unlinked with a flat prior probability distribution. We performed three independent runs of four Metropolis-Hastings Coupled Monte Carlo Markov chains (MCMCMC), three hot and one cold. Each chain was run for 1,000,000 generations and trees were sampled every 1,000 generations. Posterior probability values for the resulting 50% majority rule consensus tree were estimated after discarding the first 10% of trees as burn-in.

The final 50% majority-rule consensus tree of this analysis is presented in Figure S2A. As in other phylogenies of this group, internal branch lengths are extremely short, indicating simultaneous or near-simultaneous divergence from a common ancestor [S6-

S9]. Although this tree exhibits numerous polytomies, the resolved nodes generally have high posterior probability support.

Dealing with uncertainty. Several authors have addressed the problem of accounting for uncertainty in the phylogenetic relationships among taxa in comparative analyses [S10-S14]. Due to the rapid radiation of the Lake Malawi cichlid species flock, and the lack of a clear, species-level phylogeny for this group [S6-S9], all polytomies were generally assumed to represent true simultaneous or near-simultaneous speciation events (e.g., "hard" polytomies). When the assumption of hard polytomies holds, there should be no inflation in Type I error rates [S11]. However, in order to account for the possibility of inflated Type I error rates due to over estimation of the true number of degrees of freedom from unresolved nodes, we specified short (0.25) branch lengths for all taxa emanating from polytomies and long (1.0) branch lengths for all taxa emanating from a bifurcation in each of our analyses. This correction effectively weights the results of contrasts between taxa separated by a bifurcation while reducing the influence of contrasts between taxa from a shared polytomy [S15]. The end result is a more conservative analysis akin to a reduction in the degrees of freedom for each polytomy when calculating significance values.

Phylogenetic studies that used very large numbers of nuclear gene markers have been able to resolve the Lake Malawi phylogeny with high statistical support at the genus level [S8, S16, S17]. These studies consistently found support for morphologically defined genera; however, the true phylogenetic relationships between genera remain unknown, and no large-scale study of both mbuna and non-mbuna genera are currently

available. To take this into account, we calculated nested ANOVAs, nesting species in genus and genus in clade, using only Lake Malawi genera of which we had sampled at least two species (Figure S2D). This approach is highly conservative because it makes no assumptions about phylogenetic relationships above the genus level, and assumes that each feeding style has evolved just once within each genus. Using this highly conservative approach, we found strong trends for associations between single cone sensitivities and feeding mode, consistent with our previous analysis. (Nested ANOVA, $F_{19,12} = 2.339$, $P = 0.068$). Nested ANOVA was implemented in SPSS v17.0.

Supporting References

1. Kocher T, Conroy J, McKaye K, Stauffer J, Lockwood S (1995) Evolution of the ND2 gene in East African cichlids. Mol Phyl Evol 4: 420-432.
2. Lee W, Conroy J, Howell W, Kocher T (1995) Structure and evolution of fish mitochondrial control regions. J Mol Evol 41: 54-66.
3. Katoh K, Toh M (2005) MAFFT version 5: improvement in accuracy of multiple sequence alignment. Nucleic Acids Res 33: 511-518.
4. Ronquist F, Huelsenbeck JP (2003) MrBayes 3: Bayesian phylogenetic inference under mixed models. Bioinformatics 19: 1572-1574.
5. Nylander J, Ronquist F, Huelsenbeck JP, Nieves-Aldrey J (2004) Bayesian phylogenetic analysis of combined data. Syst Biol 53: 47-67.
6. Moran P, Kornfield I (1993) Retention of an Ancestral Polymorphism in the Mbuna Species Flock (Teleostei: Cichlidae) of Lake Malawi. Mol Biol Evol 10: 1015-1029.
7. Parker A, Kornfield I (1997) Evolution of the Mitochondrial DNA Control Region in the mbuna (Cichlidae) Species Flock of Lake Malawi, East Africa. J Mol Evol 45: 70–83.
8. Albertson RC, Markert JA, Danley PD, Kocher TD (1999) Phylogeny of a rapidly evolving clade: The cichlid fishes of Lake Malawi, East Africa. Proc Natl Acad Sci USA 96: 5107–5110.
9. Won Y-J, Wang Y, Sivasundar, A, Raincrow J, Hey J (2006) Nuclear gene variation and molecular dating of the cichlid species flock of Lake Malawi. Mol Biol Evol 23: 828-837.

10. Grafen A (1989) The phylogenetic regression. Phil Trans R Soc Lond B 326: 119-157.
11. Martins EP, T. Garland J (1991) Phylogenetic analyses of the correlated evolution of continuous characters: a simulation study. Evolution 45: 534-557.
12. Purvis A, Theodore Garland J (1993) Polytomies in Comparative Analyses of Continuous Characters. Syst Biol 42: 569-575.
13. Losos JB (1994) An approach to the analysis of comparative data when a phylogeny is unavailable or incomplete. Syst Biol 43: 117-123.
14. Garland T, Jr., Díaz-Uriarte R (1999) Polytomies and phylogenetically independent contrasts: an examination of the bounded degrees of freedom approach. Syst Biol 48: 547-558.
15. Loh Y-HE, Katz LS, Mims MC, Kocher TD, Yi SV et al. (2008) Comparative analysis reveals signatures of differentiation amid genomic polymorphism in Lake Malawi cichlids. Genome Biol 9: R113.
16. Allender CJ, Seehausen O, Knight ME, Turner GF, Maclean N (2003) Divergent selection during speciation of Lake Malawi cichlid fishes inferred from parallel radiations in nuptial coloration. Proc Natl Acad Sci USA 100: 14074–14079.
17. Kidd MR, Kidd CE, Kocher TD (2006) Axes of differentiation in the bower-building cichlids of Lake Malawi. Mol Ecol 15: 459–478.
