# Supplementary material for: The Eyes Have It: Regulatory and Structural Changes Both Underlie Cichlid Visual Pigment Diversity
Source: PLoS Biol. 2009 Dec 22;7(12):e1000266. doi: 10.1371/journal.pbio.1000266 (PMC2790343; doi:10.1371/journal.pbio.1000266)
Supplement: Text S1 — Variation at known spectral tuning sites. (0.02 MB PDF) [file pbio.1000266.s012.pdf]

Text S1: Known Spectral Tuning Sites

Microspectrophotometry suggests that *M. zebra* and *P. acei* have SWS1 visual pigments that differ in maximum sensitivity by 10 nm - confirming the variable spectral sensitivity of SWS1 pigments in Lake Malawi. This variation in spectral sensitivity correlates with amino acid substitutions that differ in polarity in the retinal binding pocket region of the SWS1 opsin. *M. zebra*, with a λ_{max} of 368 nm [1], has the combination of a serine at site 114, a threonine at site 160, and a threonine at site 204 (S114/T160/T204), while *P. acei*, with a λ_{max} is 378 nm [2], has an alanine at site 114, an alanine at site 160, and an isoleucine at site 204 (A114/A160/I204).

In the LWS opsin, variation from an alanine to a serine at site 164 (A164S) is known to long-wavelength shift LWS opsins [3] and variation in the LWS sequence of *P. pundamilia* has been shown to produce a visual pigment that is shorter wavelength sensitive than that of *P. nyererei* by 3 to 15 nm [4, 5] (Table S6). These findings suggest that the opsin sequence variation we observe generates functional variation in the corresponding visual pigments.

Supporting References

S1. Carleton KL, Harosi FI, Kocher TD (2000) Visual pigments of African cichlid fishes: evidence for ultraviolet vision from microspectrophotometry and DNA sequences. Vision Res 40: 879-890.

S2. Parry JWL, Carleton KL, Spady T, Carboo A, Hunt DM et al. (2005) Mix and match color vision: tuning spectral sensitivity by differential opsin gene expression in Lake Malawi cichlids. Curr Biol 15: 1–6.

S3. Asenjo A, Rim J, Oprian D (1994) Molecular determinants of human red/green color discrimination. Neuron 12: 1131-1138.

S4. Carleton KL, Parry JWL, Bowmaker JK, Hunt DM, Seehausen O (2005) Colour vision and speciation in Lake Victoria cichlids of the genus Pundamilia. Mol Ecol 14: 4341–4353.

S5. Terai Y, Seehausen O, Sasaki T, Takahashi K, Mizoiri S et al. (2006) Divergent selection on opsins drives incipient speciation in Lake Victoria cichlids. PLoS Biol 4: 2244-2251.
